# Task-phase-specific dynamics of basal forebrain neuronal ensembles

**DOI:** 10.3389/fnsys.2014.00174

**Published:** 2014-09-24

**Authors:** David Tingley, Andrew S. Alexander, Sean Kolbu, Virginia R. de Sa, Andrea A. Chiba, Douglas A. Nitz

**Affiliations:** Department of Cognitive Science, University of California, San DiegoSan Diego, CA, USA

**Keywords:** acetylcholine, attention, basal forebrain, parietal cortex, noise

## Abstract

Cortically projecting basal forebrain neurons play a critical role in learning and attention, and their degeneration accompanies age-related impairments in cognition. Despite the impressive anatomical and cell-type complexity of this system, currently available data suggest that basal forebrain neurons lack complexity in their response fields, with activity primarily reflecting only macro-level brain states such as sleep and wake, onset of relevant stimuli and/or reward obtainment. The current study examined the spiking activity of basal forebrain neuron populations across multiple phases of a selective attention task, addressing, in particular, the issue of complexity in ensemble firing patterns across time. Clustering techniques applied to the full population revealed a large number of distinct categories of task-phase-specific activity patterns. Unique population firing-rate vectors defined each task phase and most categories of task-phase-specific firing had counterparts with opposing firing patterns. An analogous set of task-phase-specific firing patterns was also observed in a population of posterior parietal cortex neurons. Thus, consistent with the known anatomical complexity, basal forebrain population dynamics are capable of differentially modulating their cortical targets according to the unique sets of environmental stimuli, motor requirements, and cognitive processes associated with different task phases.

## Introduction

The basal forebrain (BF) of the mammalian brain is composed of several subcortical nuclei, bearing efferents which modulate responsiveness of their neocortical targets to their cortical and/or thalamic inputs (Bigl et al., 1982; Mesulam et al., 1983; Saper, 1984; Hasselmo and Barkai, 1995; Zaborszky et al., 1997, 1999; Disney et al., 2007; Goard and Dan, 2009; Bhattacharyya et al., 2013). The complexity of this projection system is relatively high in at least two ways. First, modulation of cortical responsiveness by BF can be mediated by at least three neurochemical forms, as projection neurons may be acetylcholinergic, glutamatergic, or GABAergic (Brückner et al., 1994; Metherate and Ashe, 1995; Gritti et al., 1997; Zaborszky et al., 1999; Riedel et al., 2002; Sarter and Bruno, 2002; Nelson et al., 2005; Henny and Jones, 2008). In turn, each neurochemical system maintains a unique set of synaptic targets within any cortical region with, for example, the GABAergic projection predominantly synapsing upon GABAergic interneurons of the neocortex (Kawaguchi, 1997; Xiang et al., 1998; Porter et al., 1999; Christophe et al., 2002; Henny and Jones, 2008). Second, different BF nuclei, and even different individual neurons in any one nucleus, innervate different sub-regions of neocortex (Bigl et al., 1982; Rigdon and Pirch, 1984, 1986; Kristt et al., 1985; Zahm et al., 1996; Zaborszky et al., 1999, 2013). Because of this, there is potential for heterogeneous influence, across the cortical surface and across time, in the modulation of cortical neuron responsiveness by BF neurons. This has been demonstrated directly in a neurophysiological experiment wherein differential modulation of auditory and visual cortices to auditory and visual stimuli resulted from activation of neighboring BF neurons (Golmayo et al., 2003). Anatomical specificity has also been observed in the activation of prefrontal cortex-projecting vs. motor cortex-projecting BF acetylcholine (ACh) neurons during task performance (Parikh et al., 2007).

Given the complexity and the anatomical reach of the BF projection systems, it is perhaps not surprising that both cell-type-specific and non-specific lesions of BF yield impairment in attention (Muir et al., 1993; Chiba et al., 1995; Stoehr et al., 1997; McGaughy et al., 2002), learning and memory (Biggan et al., 1991; Baxter et al., 1995; Baxter and Chiba, 1999; Leanza et al., 1996), cortical desynchronization (Szymusiak and McGinty, 1986), and cortical plasticity (Zhu and Waite, 1998; Conner et al., 2003; see also Roberts et al., 1992). Yet the neurophysiological form by which BF neurons participate in such processes remains largely unknown, primarily for lack of extensive data concerning the dynamics of BF neurons during performance of complex tasks wherein different combinations of environmental stimuli, motor states, and cognitive processes (e.g., working memory, reward encoding, decision-making) occur in established sequences across task trials. Furthermore, the available data suggest that the dynamics of BF neurons could well be limited to macro-level variables such as sleep/wake state (Buzsáki et al., 1988; Szymusiak et al., 2000; Lee et al., 2004; Goard and Dan, 2009; Hassani et al., 2009) and/or to encoding onset of a relevant stimulus and reward receipt (Wilson and Rolls, 1990a,b; Parikh et al., 2007; Lin and Nicolelis, 2008; Bhattacharyya et al., 2013). In the latter case, it even appears that there is only weak distinction, for any given BF non-cholinergic neuron, in activity responses to stimuli and reward. Thus, despite the multifarious neuroanatomical organization of the basal forebrain (for review see Zaborszky, 2002; Zahm, 2006; Chiba and Quinn, 2007), the current neurophysiological picture would suggest that firing properties of BF neurons are similar to those observed for other neuromodulatory systems with cortical projections, such as the dopaminergic and noradrenergic systems, wherein most neurons respond to all target, unexpected, and/or rewarding stimuli (Aston-Jones and Bloom, 1981; Aston-Jones et al., 1991, 1994; Schultz, 1997; Bouret and Sara, 2005).

Thus, at present, it remains to be determined whether the response fields of BF neurons match their anatomical and cell-type complexity, or whether BF neuron populations operate as a relatively undifferentiated group to all salient and reward stimuli. We address this question in the present work, wherein ensembles of neurons in the substantia innominata and ventral pallidum sub-regions of BF were recorded during performance of a visuo-spatial selective-attention task, for which temporally distinct target detection, decision-making, and outcome evaluation phases could be defined. To assess the potential impact of BF activity patterns on their neocortical targets, we also obtained recordings from one BF target, the posterior parietal cortex (PPC), during performance of the same task (Kristt et al., 1985; Gritti et al., 1997; Zaborszky et al., 1997; Bucci et al., 1998; Nelson et al., 2005; Maddux et al., 2007; Broussard et al., 2009). This cortical sub-region is a recipient of projections from substantia innominata and ventral pallidum and lesion of its cholinergic neurons yields impairments in incrementing attention to sensory stimuli (Bucci et al., 1998). Given that PPC neurons exhibit a variety of response types during navigational tasks (e.g., Nitz, 2012), this cortical sub-region was expected to exhibit task-phase-specific firing patterns suitable for direct comparison with those of basal forebrain neurons.

We present evidence supporting the contention that BF neuron task-related activity goes well beyond simple responses to target and reward stimuli. Among the full population of BF neurons, multiple distinct and complex forms of task-phase-specific activity are realized, forming unique population firing patterns for all task phases. All of the observed task-phase-specific patterns of BF sub-groups were also observed in PPC neuron sub-populations, evidencing a potential role for BF efferents in differentially organizing cortical activity according to task demands at all points in time.

## Methods

### Subjects

All experimental protocols adhered to AALAC guidelines and were approved by IACUC and the UCSD Animal Care Program. Eight adult, male Long-Evans rats served as behavioral subjects. Rats were housed individually and kept on a 12-h light/dark cycle. Prior to experimentation the animals were habituated to the colony room and handled daily for a period of 1–2 weeks. After this period, animals were placed on food restriction until they reached 85–90% free-fed weight. Water was available continuously. Rats were required to reach a minimum weight of 350 g prior to surgery and subsequent experimentation.

### Task description and performance

**Figure 1:**
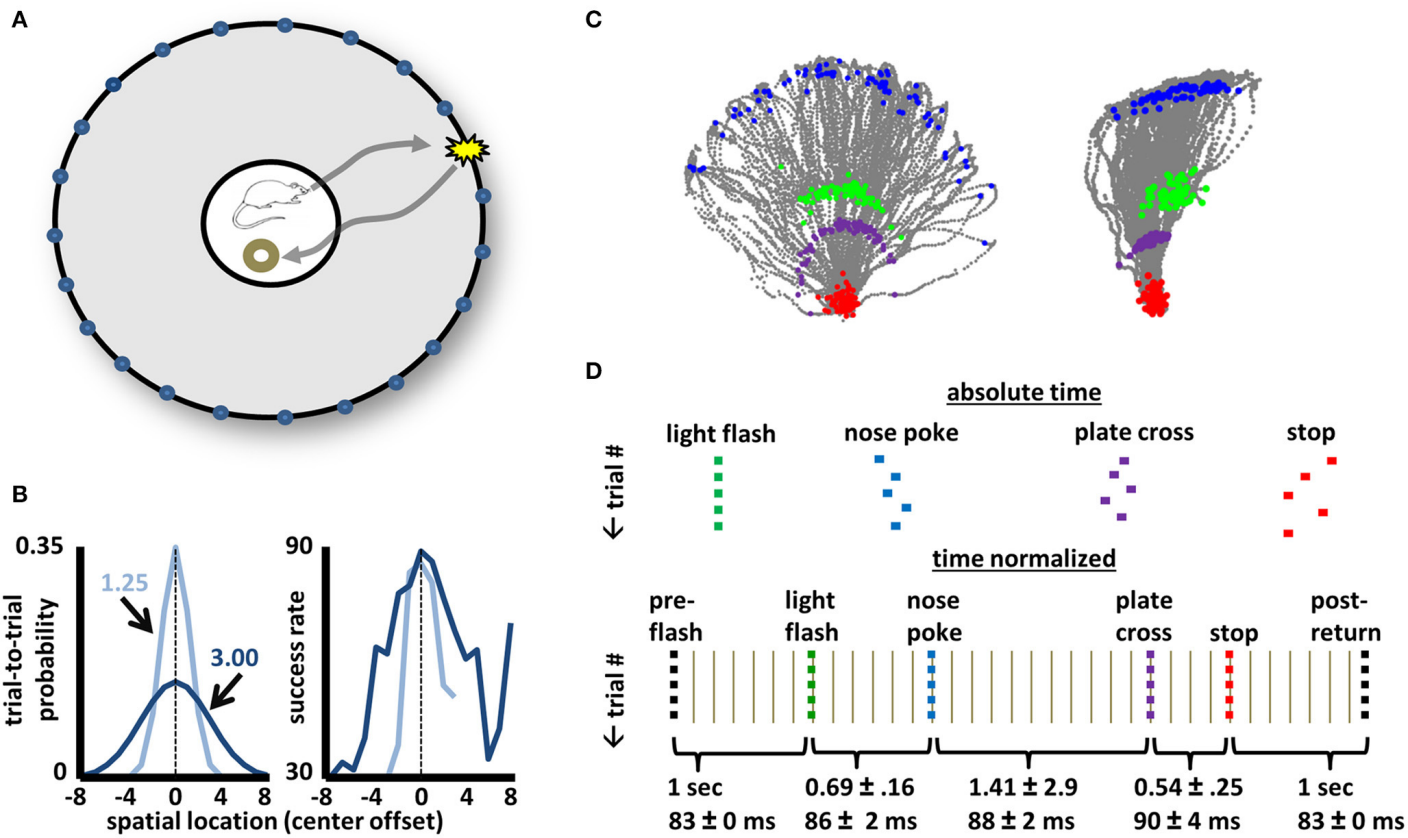
**Design, performance, and task phase identification for a selective-attention task. (A)** A schematic of the arena used for the selective attention task. A 1.2 m-diameter environment with 46 cm walls having 36 evenly-spaced light sources each 6.5 cm above the surface. A trial begins when the animal stands upon the 25 cm plate at the arena center with head orientation such that the location of any individual trial's light flash lies within a 120-degree space centered on the animal. A light flash (150 ms) from a single location triggers a journey to identify the spatial location of the flash with a nose-poke. Return to the center plate yields 1/2–piece Cheerio reward if the correct light source was identified. **(B)** Left panel: Two trial-by-trial probability distributions (Gaussians with 1.25 or 3.0 *SD*) from which the light source locations were chosen randomly for any given animal/recording. Right panel: Average success rates for all animals across all recording sessions approximately match the shape of the light-source probability distributions. **(C)** Tracking data for all correct trials for recordings utilizing the medium (left image) and narrow (right image) probability distributions of **(B)**. For each trial, gray points map the animal's movement for time periods 1 s before light flash until 1 s after stop/reward. Green points correspond to the animal's position at the time of the light flash, blue points mark position at the time of nose-poke, purple points the crossing onto the center plate upon return, and red points the positions where the animal stopped and obtained reward. Purple points effectively outline the perimeter of the center plate (reward was delivered at the back end of the plate). Note that all animals tended to lean far over the rim of the center plate while awaiting the light flash such that head positions at the time of light flash and center plate return are non-overlapping. **(D)** A schematic of the time-normalization procedure used to account for slight cross-trial differences in time intervals between distinct task events (light flash, nose-poke, plate return, stop/reward obtainment). The normalization procedure permits neuronal dynamics across all task phases and all trials to be considered simultaneously. Intervals between identified task events (e.g., light flash to nose-poke) were ascribed a specific number of time bins, with the time of each bin adjusted on a trial-by-trial basis such that the full time interval is spread evenly across bins. The mean time (in seconds, ±STD) between major task events is given below as is the mean duration (in ms) for all time bins within each interval. The same color-coding of major task events is used in all subsequent figures.

**Figure 2:**
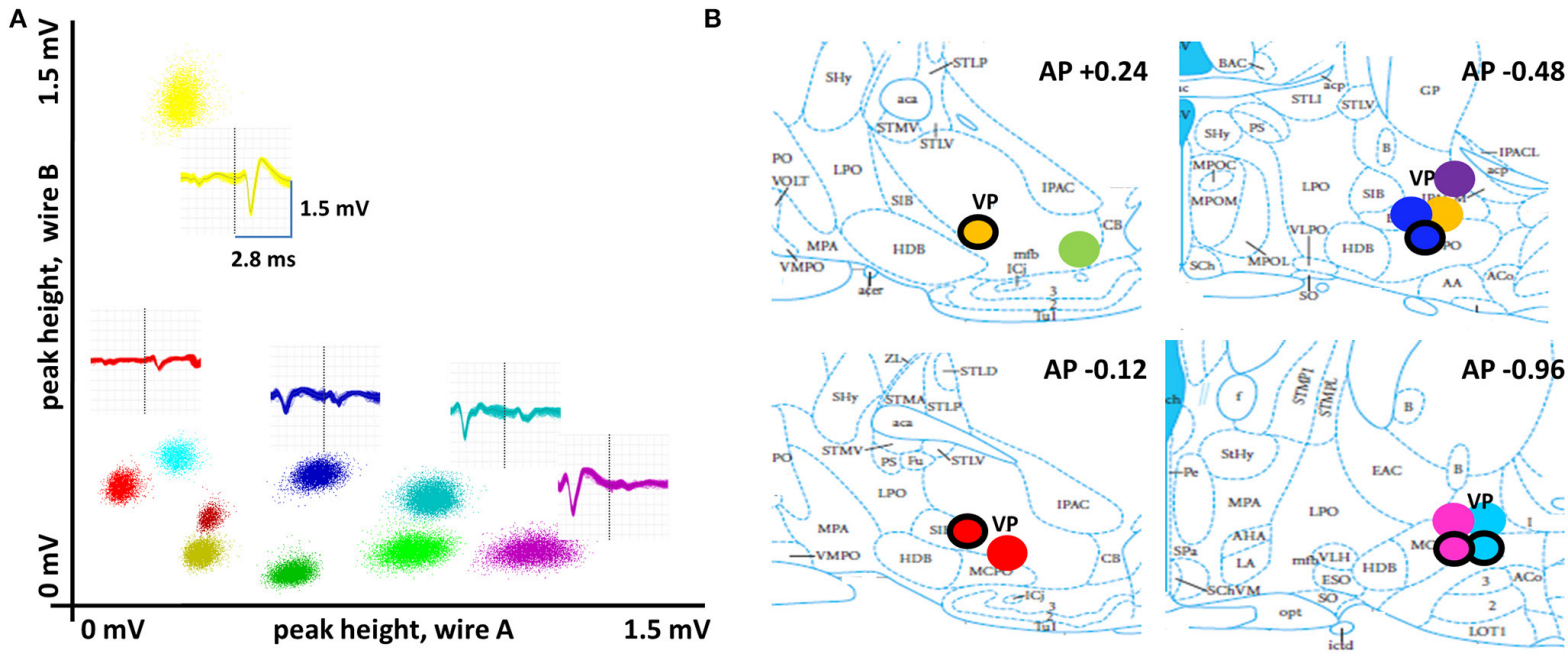
**Basal forebrain recording sites and properties of spike waveforms. (A)** Spike waveforms for multiple single neurons recorded with stereotrodes were discriminated according to their clustering along multiple dimensions of waveform parameters. Here, peak heights on each wire of a stereotrode are plotted against each other for all spikes of 10 basal forebrain neurons. An overlay of the full set of waveforms is given for five of these (vertical line separates the two wires of the stereotrode in each). **(B)** Summary of recording sites (*N* = 8 rats, 12 stereotrode bundle placements) in sub-regions ventral pallidum and substantia innominata of the basal forebrain. Differently-colored, filled circles represent different animals. Circles with a black ring represent location of recording sites for analogous positions in the left hemisphere.

In the context of a selective attention task described in Figure 1, activity dynamics of basal forebrain neurons (*N* = 1428 from 74 recordings, see Figure 2) were recorded. All animals had extensive experience (>30 daily sessions) prior to surgery. Adult male rats (strain Long-Evans, *N* = 8) were trained to perform the task within a 1.2 m diameter arena (Figure 1A). Trials (100/day) began with the animal positioning himself within a 25 cm diameter region at the arena center that was defined by a plate having a 5 cm lip. Correct performance on the task involved: (1) detection of a light flash (150 ms) from one of 36 possible light sources positioned behind nose-poke holes (6.5 cm above the arena surface) and staggered at 10° intervals along the arena perimeter; (2) travel to the arena perimeter to identify the spatial location of the light source through nose-poke; and (3) direct return to the arena center to obtain food reward (Figure 1A). The spatial position of a light flash for any given trial was randomly selected from one of two probability distributions (Figure 1B, left panel). Reward (1/4 piece Honey Nut Cheerio) was delivered manually by placement near the back edge of the center plate. Movement to place reward was begun only after determination (easily observed) of whether the animal had correctly identified the light source. On error trials (not examined in this work), the experimenter moved to place the reward in the same way, but did not release it prior to withdrawal. No reward was given on trials associated with incorrect identification of the light source. “No-Go” trials, in which the animal failed to respond to the light flash with locomotion to the arena perimeter, constituted less than 5% of all trials in all animals indicating solid performance with respect to detection of the light flash itself. However, daily success rates ranged from 26 to 93% for the recording sessions analyzed in the present dataset (mean 70 ± 13%, see Supplemental Figure 2). Furthermore, the statistical regularities in cross-trial spatial locations of light flashes impacted performance. Specifically, the spatial locations of lower-probability lights were less likely to be correctly identified (Figure 1B). Thus, the task demands attention in detecting and, in particular, locating, a brief visual stimulus and in correctly performing a series of motor responses to obtain a reward.

### Surgery

Rats were implanted with arrays of 8 stereotrodes (25 micrometer tungsten with polyimide insulation; California Fine Wire, Grover Beach, CA) built into custom-fabricated microdrives. Wire tips within arrays had a spread of approximately 3/4 mm in the medial/lateral and anterior/posterior planes. Two such microdrive arrays were implanted in each animal with two targeting left and right BF (AP 0.2 mm, ML 2.8 mm, V 7.0 mm) Another drive housing an array of 4 tetrodes (17 micrometer platinum/iridium with polyimide insulation; California Fine Wire, Grover Beach, CA) targeting right PPC (AP −4, ML 2.5, V 0.5) was used in five animals. In the remaining three animals, one stereotrode microdrive targeted right BF and two tetrode arrays targeted left and right PPC. Dorsal-ventral coordinates were chosen to permit slow movement of the recording wires into the desired BF (V 8–9) and PPC (V 0.8–1.5) target areas across days in which the animal was re-introduced to the task.

### Recordings

All electrodes were bundled into custom-built microdrives permitting movement in 40 μm increments in the dorsal-ventral axis. Recording wires were cut to the same lengths and gold-plated to reach impedances of approximately 100 kOhms (Tungsten stereotrodes) and 300 kOhms (Platinum/Iridium tetrodes). All recordings were made using the Plexon MAP system (Plexon, Dallas, TX). Signals were amplified at the level of the headstage connection (20×), again at a pre-amp stage (50×), and then to varying degrees, as appropriate, at the amplifier stage (additional 1–15×). Unit signals were bandpass filtered (450–8.8 KHz). Candidate spike waveforms (exceeding an amplitude threshold) were recorded using SortClient (Plexon, Dallas, TX) at a sampling frequency of 40 kHz.

Waveform discrimination into individual units was carried out manually using Plexon's OfflineSorter software. All waveform discrimination in the present work was undertaken by one highly-trained technician (trained and closely supervised by corresponding author Nitz) to maintain stability in the approach. The process begins by identifying collections (clusters) of waveforms that share the same relative spike amplitudes for the two wires of any given stereotrode. To avoid identification of neurons for which only a portion of all spikes have been collected, waveform clusters whose lowest-amplitude members are near the amplitude threshold are excluded. Subsequent to the initial assignments of waveforms into clusters, further refinement takes place using a variety of waveform parameters. Such parameters include valley depth and energy, but also include the “score” of each waveform for its similarity to each of several principal components defined objectively by the Plexon software based on the full set of waveforms. These parameters permit refinement of clustering through removal of recording artifacts and by identification of instances in which two neurons share the same relative spike amplitudes for both wires of a stereotrode, but have no overlap with respect to other waveform parameters (i.e., have differently shaped action potentials). Two procedures were implemented to account for the relatively rare cases in which waveform amplitudes varied in a systematic way across the time of a full recording: (1) During the waveform discrimination process, we examine spike amplitudes for each neuron as a function of recording time. In those instances where spike amplitudes vary considerably or in a way that might generate confusion in discriminating two neurons, we simply exclude the cell(s) from further analysis; (2) We completed a full analysis of every neuron's firing rate across individual trials, allowing us to identify and remove those very few neurons for which extreme changes in firing were found (typically this corresponds to a neuron completely shutting off or turning on at some point in the middle of the recording).

The animal's position within the environment was detected from overhead images of the arena at 60 Hz. using Plexon's CinePlex Studio. Tracking software picked up light from two differently-colored LEDs clipped to a connector embedded in the dental acrylic used to fix microdrives to the animal's skull.

Stereotrode bundles were adjusted across days as necessary to maintain collection of large numbers of high-amplitude action potential waveforms (as many as 60 per day). Data included in the present set of analyses were, for all individual animals, associated with different depths (minimum 80 μm separation) to greatly minimize the possibility that single neurons could contribute to the full dataset more than once. Supplemental Table 1 lists the animal subject, cell count, and probability distribution's standard deviation for each of the 74 recordings.

### Behavioral event analysis

Position tracking data was analyzed using a custom Matlab (Mathworks, Natick, MA) guided user interface. Each trial was closely examined to identify the position point associated with initial movement to the light source, and the sharp point of trajectory reversal associated with nose poke. The time points at which the animal crossed back over the perimeter of the center plate and at which the animal stopped to consume reward were determined through automated analysis of positional data using Matlab. Trials in which the animal did not make ballistic, direct runs to and from the site of a nose-poke were not included so that trial-to-trial variability in task epoch durations were kept minimal relative to task epoch mean durations.

### Time-normalization and firing rate calculation

To enable characterization, categorization, and comparison of neuronal activity across all trials and all behavioral epochs, we utilized a time normalization procedure to align neural data for light-onset, nose-poke, center plate return, and stop/reward times. Time normalization was accomplished by identifying the average time between light flash to nose-poke, nose-poke to center return, and center return to stop/reward across all trials and animals. On average it took the rodent 0.69 s to reach the light-port after the light flash. Animals took a mean of 1.41 s to return to the plate after nose poke and 0.54 s to stop to consume reward after having crossed onto the center plate. We divided these periods into ~80–100 ms time bins for each trial. There are slight deviations from these averages for all animals across trials, thus, the bin duration was allowed to fluctuate slightly in order to allow for the behaviorally significant events to consistently occur at the same bin. A 1 s period before light-flash and after stop/reward was included in each trial to include stimulus expectation and reward consumption time periods, respectively. By this process, we obtained vectors of time-normalized data in which a pre-light flash period composed bins 1–12, light-flash to nose-poke in bins 13–20, nose-poke to center plate return in bins 21–36, center plate return to stop/reward in bins 37–42, and a post-trial reward period in bins 43–54. All firing rate vectors illustrated in this paper are in this 54-bin format. Mean firing rate vectors were calculated for each cell on each recording day across all trials and for each bin of normalized time. All data and analyses presented are based solely on rewarded trials (i.e., those associated with choice of the correct light source). To permit optimal visualization of BF firing patterns, normalized firing rates are presented in most figures. Such normalization is made on a cell by cell basis by finding the task-phase specific firing rate bin having the highest value and thereafter expressing that and all other firing rates as a proportion of that highest value. Thus, every neuron depicted has at least one firing rate bin with a value of 1 (the time bin associated with peak firing).

### Noise correlation

Deviations from the mean firing-rate vector for all task phases were obtained for each individual trial, for each recorded neuron. Deviation vectors for all trials were appended in series and a Pearson correlation was calculated for pairs of these vectors for all simultaneously recorded neurons. This is referred to as the “noise” correlation (Zohary et al., 1994; Kargo et al., 2007). For comparison, a boot-strapped control was generated for each neuron pair by randomly shuffling trial orders for one neuron of a pair to determine the mean noise correlation expected by chance (*N* = 100 randomizations). “Signal” correlation is defined as the Pearson correlation between the mean, cross-phase firing rate vectors for any two neurons. Neuron pairs recorded on the same vs. different stereotrodes were considered separately with the result that the mean absolute value of noise correlation was 0.10 ± 0.05 (STD) for same-stereotrode pairs and 0.11 ± 0.06 for different-stereotrode pairs. Given the marginal difference, data were pooled.

### Histology

Animals were perfused with 4% paraformaldehyde under deep anesthesia. Brains were removed, sliced into 50 μm sections using a sliding microtome, and Nissl stained. The point of deepest electrode penetration was used in conjunction with microdrive adjustment records to determine the range of depths sampled for any given stereotrode bundle placement. The spread of electrodes within an array rendered it difficult to clearly determine which individual stereotrodes were within vental pallidum vs. substantia innominata and so separate analyses of spiking activity within these structures was not possible.

## Results

### Ensemble firing patterns of basal forebrain neurons distinguish all task phases

One goal of the present work was to examine BF neuron activity patterns in relation to multiple phases of a selective attention task (Figure 1). The merit of this approach lies in the ability to better identify potential complexities in the response fields of BF neurons (e.g., multiple and/or sustained firing peaks at and across task phases) that cannot be achieved through examination of peri-event histograms. To this end, the task was designed to identify precise time-points associated with temporally punctate stimuli and actions such as light flashes, nose-pokes, and reward obtainment (Figure 1C). The structure of this task also generated temporal space between such events, as well as regularity and measurability in motor behavior separating them. Thus, constraints were placed on the positioning and orientation of the animal prior to onset of the light flash, training promoted ballistic motion of the animal (i.e., uninterrupted center-out and return runs), tracking data enabled deletion of trials associated with atypical behavior, and a “time-normalization” procedure was employed to account for slight trial-to-trial differences in time intervals between major task events (Figure 1D).

**Figure 3:**
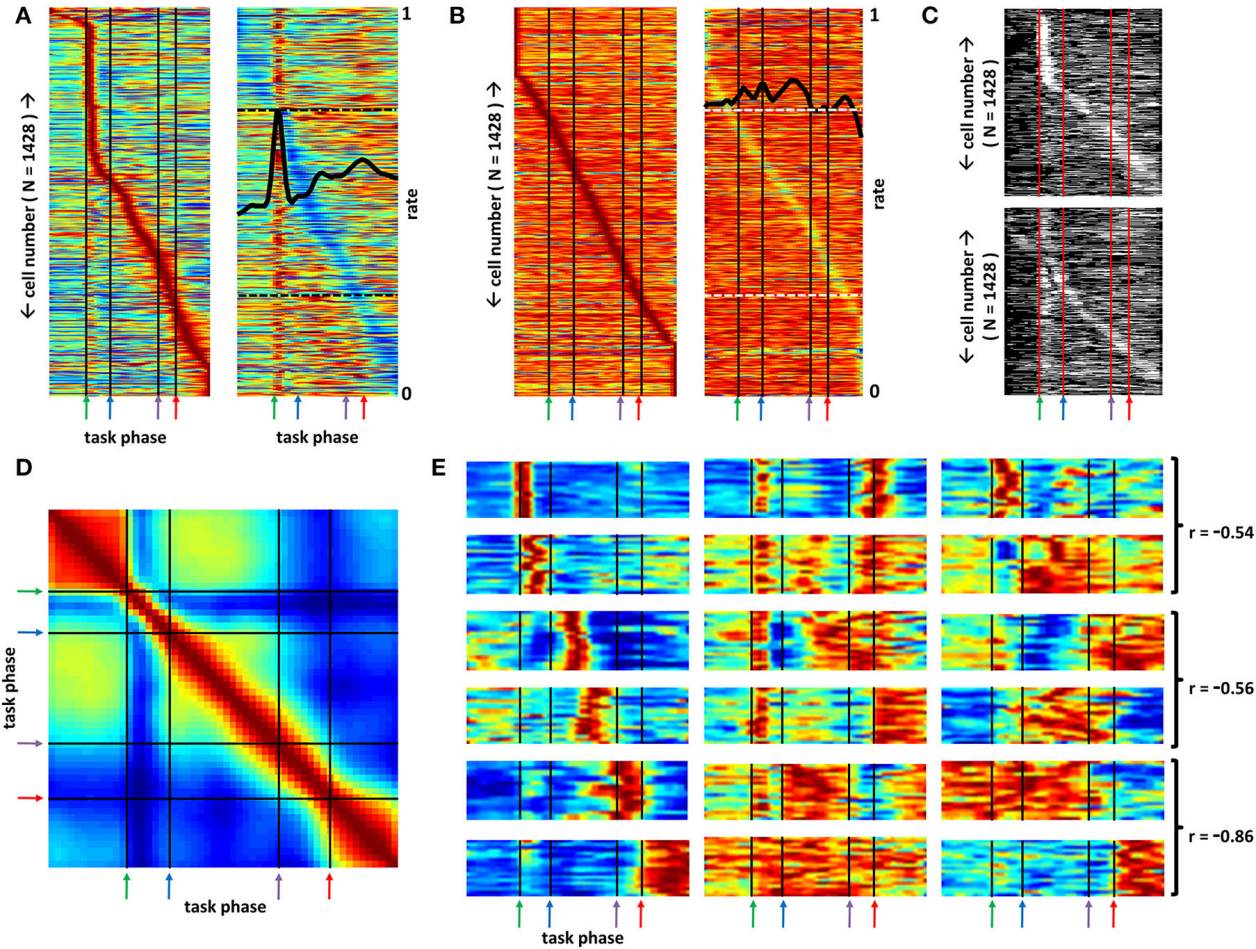
**Distinct sub-populations of basal forebrain neurons distinguish all phases of a selective attention task**. **(A)** Left panel: Mean firing rates, each normalized to their own in-task maximum rate (blue-red = 0–1), of all recorded BF neurons (y-axis) across all phases of the task (x-axis). Green, blue, purple, and red arrows demark, respectively, the light flash, nose-poke, plate-cross and stop/reward phases of the task. Neurons are ordered according to the task phase associated with peak firing rate to demonstrate that large populations of neurons fire at their maximal rates for every task phase. Right panel: Maximum-normalized rate vectors for the same neuron population, but organized according to the task phase associated with lowest rate. The mean, peak-normalized firing rate of all neurons is given by the overlaid black trace (see right side of panel for scale; dashed black lines mark 0.25 and 0.75 on this axis). This rendering permits visualization of the multiple peaks and troughs in firing observed for many neurons. **(B)** Mean maximum-normalized firing rate vectors for the same set of neurons as in **(A)**, but generated using randomly chosen light-onset times (blue-red = 0–1). The full set of rate vectors has been reordered according to the task phase at which neurons exhibit their peak (left panel) and trough (right panel) firing rates (early peak neurons at top, late peak neurons at bottom) such that individual neurons in **(A)** and **(B)** will occupy different positions on the associated y-axes. Under these conditions, the maximum mean firing rates observed are much closer to the mean rates across all task phases such that these panels contain many more values near the firing rate maximum (i.e., more orange and red bins). Left panel and right panel depict the same data organized according to time of peak and trough firing, respectively. Black trace in right panel depicts mean of all neurons for each task phase (see right side of panel for scale; dashed white lines mark 0.25 and 0.75 on this axis). **(C)** Statistical significance (white) or lack thereof (black) for every neuron's firing rate at each task phase. Neuron order (y-axis) for the top panel follows that of the left panel of **(A)** and that for the bottom panel follows that of the right panel of **(A)** to depict significance for most firing rate peaks and troughs as well as a large proportion of off-peak and off-trough mean firing rates. **(D)** Correlation matrix for the data of **(A)** yields little pattern recurrence indicating that BF firing patterns are unique for every task phase. Each value reflects the Pearson correlation between the ensemble firing rate vectors for all cells at the task phases associated with that bin's X and Y axis positions (blue-red = 0–1). **(E)** Each panel depicts maximum-normalized mean cross-trial firing rates (color axis 0.2–1) for sets of individual BF neurons (y-axes) that share task-phase-specific activity patterns as determined by clustering analysis (see Section Methods). Some neurons exhibit significant rate change for only a single task phase such as time of light flash, travel to the nose-poke, the middle of the return trip to arena center, the time of center plate crossing, and the time at which the animal stops and obtains reward (column 1, rows 1–6 respectively). For other neuron categories multiple peaks and/or troughs in firing are observed (col. 2, rows 1–5) and firing may persist across multiple time bins (col. 2, rows 2–6). For many categories, a parallel, but opposite pattern of activity is observed compared to that for other categories. Three examples of such pairs (joined by brackets) are given in the third column along with the Pearson correlation of the mean patterns for each.

Figure 2 describes features of the action potential waveform discrimination process (see also Methods) and depicts the histologically-determined recording sites for all animals. Task-phase-specific mean firing rate vectors for the full population of BF neurons were first organized according to the task phases associated with maximal and minimal activity (Figure 3A), revealing basic properties of BF function not predicted by prior work. First, distinct subsets of BF neurons reach peak activity at each task phase, including, for example, the time period directly between nose-poke and return to the center plate. This indicates that BF function is not limited to registering time of reward obtainment and/or the onset of sensory stimuli having acquired salience. Rather, BF population dynamics are consistent with a model in which BF differentially impacts activity and responsiveness in its efferent targets at all points in time, in an organized manner. Additionally, at all task phases, the population of most-active BF neurons is paralleled by a population exhibiting temporally precise activity troughs (typically zero firing).

To examine the statistical significance of the observed firing rate fluctuations in Figure 3A, bootstrap control firing rate vectors were generated by assigning random light-onset times for each neuron and for each trial. For these random-time trials, intervals between light onset, nose-poke, plate-crossing, and reward-obtainment times were based on the intervals observed for the non-randomized dataset. The left and right panels in Figure 3B depict mean firing rate vectors from this control dataset, organized by time of peak and trough firing rates, respectively. *T*-tests (alpha set at 0.01) were then performed, comparing the cross-trial firing rates for each neuron at each task phase for the actual data against the cross-trial firing rates for the corresponding neuron/phase of the control dataset. Figure 3C depicts the results of these tests. White pixels reflect those neuron/task phase combinations associated with statistically elevated or decreased firing rates. Across all neurons, 82.8 and 62.2% of peak and trough firing rates, respectively, reached statistical significance. Across all neurons and task phases, fully 33.8% of the observed mean firing rates yielded significance, a value far above that expected (1%) as well as that observed (1.02%) for the same set of comparisons for two separate randomizations (data not shown). Critically, this percentage reflects statistical significance for many of the off-peak and/or off-trough firing rates, indicating that many neurons exhibit multiple task-phase-specific changes in firing rate.

These results indicate that BF function must be considered according to the full pattern of activity among BF neuron populations and that depressions in BF activity may have as great an impact on efferent targets as increases in activity. Such consideration is given in Figure 3D, where recurrences in BF neural patterns across task phase were detected by correlating the ensemble firing pattern at any given task phase with those at all other phases. While persistence in BF ensemble firing patterns is found over the time period preceding light flash onset and after reward obtainment, further evidence for pattern recurrence is not found, directly evidencing the fact that BF networks are capable of generating a large repertoire of distinct firing patterns. Finally, the organizational scheme applied in Figure 3A (right panel) reveals that a large proportion of BF neurons have multiple activity peaks and troughs and may, in some cases, exhibit activity persisting across several task phases. That is, response fields for BF neurons maintain a high degree of complexity.

### Defining categories of task-phase-specific rate vectors

The apparent complexity of BF neuron task-phase-specific responses suggests that the classical approach, that is, determining proportions of individual neurons responding to individual stimuli (e.g., light flash) or actions (e.g., nose-poke), will lead to some degree of oversimplification in revealing the response fields of BF neurons. Instead, a custom, unsupervised clustering algorithm was applied to the full array of task-phase-specific mean firing rates. In brief, the algorithm consisted of three steps: (1) dimensionality reduction of the full array using principal components analysis; (2) initialization of clusters with K-means (500 restarts, *K* = 60); and (3) refinement of clusters according to a Gaussian mixture model wherein the full covariance matrix in the reduced space was utilized. The first eight principal components were utilized for clustering and explained 93% of the variability in task-phase-specific firing patterns (Supplemental Figure 3). Notably, while very useful in enhancing visualization of recurring patterns within complex datasets, the categorization analysis, as used, is not intended to define a specific number of “true” categories of BF neurons, nor to define their neurochemical identity. Instead, it is intended to identify the structured patterns of the basal forebrain as they directly relate to the ongoing behavior of the rat.

Categorization of the population of mean rate vectors revealed many forms and correlates of BF spiking activity not previously described, most of which were observed in many or all of the animal subjects (Supplemental Figure 1). For example, mean firing rates for BF neurons falling into 18 different groupings are given in Figure 3E (please see Supplemental Figures 1 or 3 for the full set). Several groups have neurons exhibiting sharp increases in firing across a single task phase, but, across groups, that task phase may differ greatly (Figure 3E, left column). As expected (Wilson and Rolls, 1990a,b; Tindell et al., 2004; Lin and Nicolelis, 2008), firing peaks corresponding to light-flash onset and reward obtainment are found, but responses specific to time periods associated with movement to or from the arena perimeter are also seen (Figure 3E, left column). Thus, specific groups of BF neurons exhibit response fields that, as a sequence, parallel the changes in sensory input, motor output, and cognitive processing that occur across task phases.

**Figure 4:**
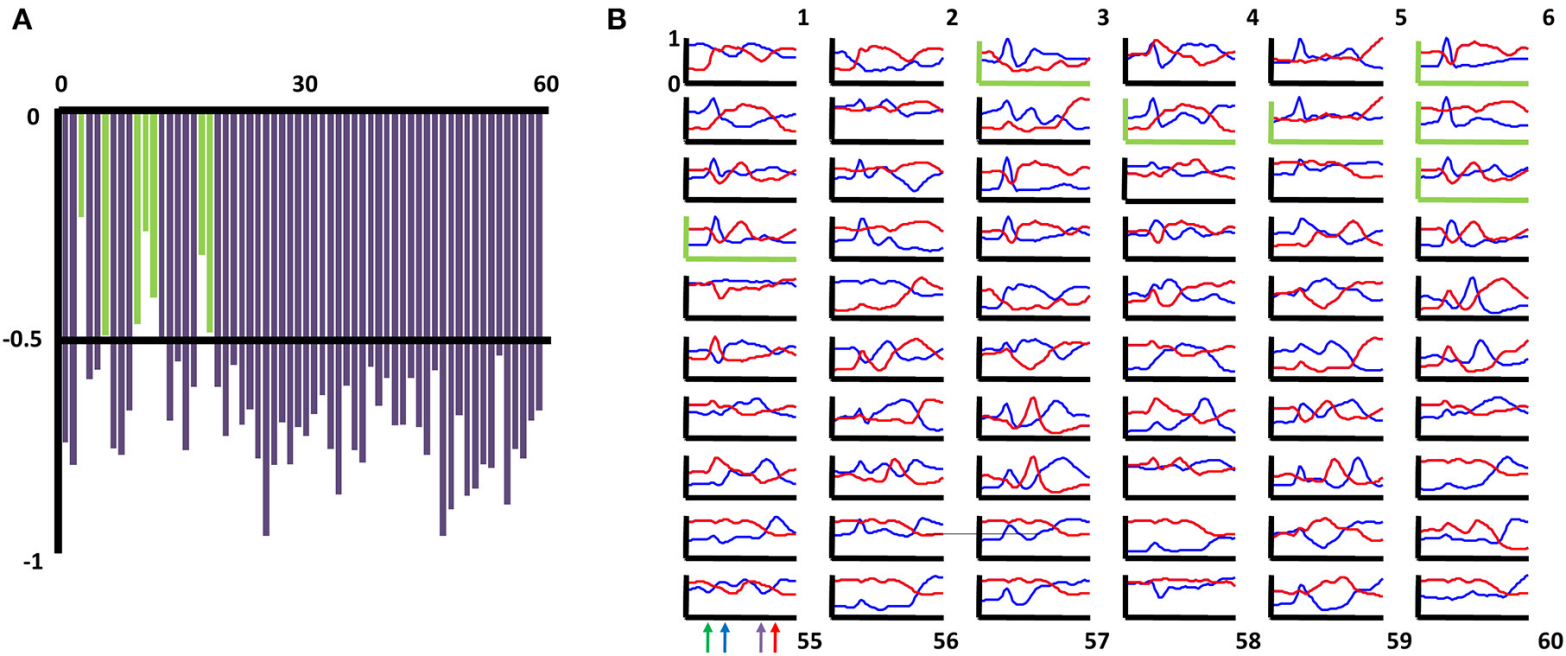
**Basal forebrain categories of task-phase-specific firing have counterparts with negatively correlated patterns. (A)** For each of the 60 categories of task-phase-specific basal forebrain (BF) firing patterns, the correlation of the mean firing pattern for that group's members (neurons) and all other groups was determined. Shown are the maximally negative correlations obtained for each group. 53 of the 60 groups (88%) had counterparts for which the mean firing pattern among its members bore a correlation more negative than (−0.5). Those yielding weaker correlations are shown as green bars (categories 3,6,10,11,12,18,19). **(B)** For each of the 60 categories of task-phase-specific basal forebrain firing patterns, the mean firing pattern for that grouping of neurons is given by the blue traces (x-axis task phase, y-axis max-normalized firing rate). Red traces reflect the mean firing pattern for the category whose mean firing pattern yielded the most negative correlation. Graphs with green axes correspond to those categories in **(A)** for which no correlation more negative than −0.5 was obtained.

The categorization analysis also reveals great complexity and heterogeneity in the response fields of BF neurons. BF neurons having dual responses to relevant light flashes or auditory tones as well as reward delivery have previously been observed (Wilson and Rolls, 1990a,b; Tindell et al., 2004; Lin and Nicolelis, 2008). However, consideration of all task phases revealed many more categories of neurons exhibiting multiple peaks in firing (Figure 3E, middle column), and, again, most of the patterns were observed in most of the animals recorded (Supplemental Figure 1). Rate vectors associated with 2–3 peaks in firing rate are common across the full population, and the specific sets of task phases associated with such peaks differ greatly across neuron groupings. BF neuron responses can be transient, consistent with responses to specific task-related stimuli, but may also persist, seemingly driven by sensory, motor, or cognitive processes that persist across an extended series of task phases. Finally, the categorization analysis revealed sub-populations of BF neurons that are oppositely tuned across all task phases. The mean task-phase-specific firing pattern for nearly every grouping of BF neurons has a counterpart in another grouping for which the mean task-phase-specific pattern is strongly negatively correlated (Figure 3E right column). This was revealed by an analysis in which the mean task-phase-specific firing rate vector for each category was correlated with that for all other categories. Figure 4A depicts the maximally negative correlation obtained for each category against all others. In all but a few cases, correlations less than −0.5 are observed. Figure 4B depicts the mean rate vector for each category against that for the category with which it was most negatively correlated.

**Figure 5:**
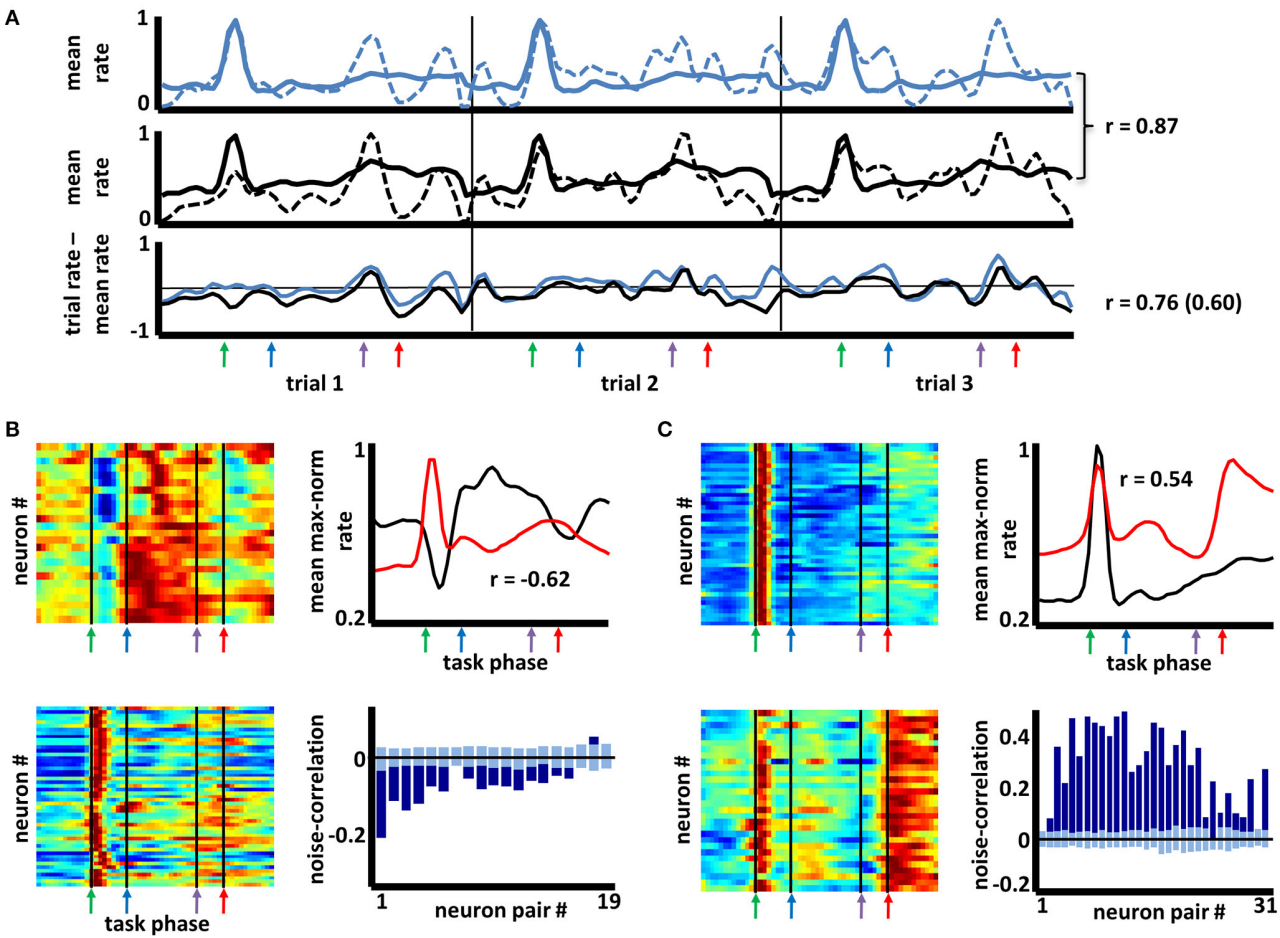
**Noise correlation among basal forebrain neurons having similar or dissimilar firing patterns. (A)** Upper and middle panels depict max-normalized firing rates for two simultaneously-recorded basal forebrain neurons across three consecutive trials (dashed lines in each; thin vertical lines separate trials). Thick lines depict, in repeating fashion, the cross-trial firing rate means for the same two neurons. The mean firing patterns for the two neurons are strongly correlated (*r* = 0.87). Lower panel depicts subtractions of each neuron's single trial rates from their cross-trial mean rates. The deviations from the mean for the two neurons are correlated across the three trials (*r* = 0.76) as well as across all trials (*r* = 0.60). **(B)** Color maps depict task-phase-specific firing rates for individual members of two categories of neurons (color axis 0.2–1). The mean rates for each category (upper right panel) bear a strongly negative correlation (i.e., a negative “signal” correlation, *r* = −0.62). Pairs of simultaneously recorded neurons from these groups show a bias toward negative correlation (lower right panel, dark blue bars) in their trial-to-trial deviations from mean, task-phase-specific rate (i.e., negative “noise” correlations). Light blue bars reflect mean (±2X STD) noise correlations when trial order was randomly shifted for one neuron (*N* = 100 randomizations). **(C)** Similar plot to **(B)** but with two groups of BF neurons that have a positive signal correlation (*r* = 0.54). Positive signal correlations among any pair of neurons typically yielded positive noise correlations far greater than those expected by chance (color axis 0.2–1).

The foregoing results suggest that, for complex behavioral tasks, BF neurons are organized into distinct groups by virtue of the anatomical organization of their local interconnections, by their extrinsic afferents, or both. Irrespective of whether such patterning reflects learning processes, if such a highly organized pattern of connectivity exists, then variation in task-phase-specific firing rates for any pair of neurons should tend to follow the degree to which their mean firing patterns are similar or dissimilar. Evidence for this was obtained in a “noise correlation” analysis summarized in Figure 5. Noise correlation determines, for any two neurons, the extent of any relation between their respective deviations from mean firing rates on each trial (and, in this case, for each task phase; Zohary et al., 1994; Kargo and Nitz, 2004). In the example given in Figure 5A, the task-phase-specific firing rates for two neurons across three succeeding trials are appended across the x-axis (dashed lines in upper and middle panels, thin vertical lines separate trials). For comparison, the mean cross-trial firing rates for the same neurons are repeated (full lines). The lower panel depicts the subtraction of these cross-trial mean rates from the firing rates for each phase of each individual trial. For this pair of neurons, the mean firing rate patterns are highly similar (*r* = 0.87) as are patterns of deviation from their means (*r* = 0.76 for the trials shown, *r* = 0.60 across all trials). That is, the spike rate deviations from mean cross-trial rates are not stochastic, but reflect co-variation of the neuron pair's spiking responses.

For all neuron pairs having significant correlation in their mean firing rate vectors (“signal” correlations >0.345 or <−0.345 using 54-bin rate vectors), the statistical significance of their noise correlation was examined by comparing it with that obtained when trial orders were shuffled for one of the two neurons. For each pair, the noise correlation expected by chance was taken as the mean of 100 such randomizations of trial identity. Across 14,020 pairs having positive signal correlations, 62% exhibited noise correlation values two standard deviations or more greater than that expected by chance. Significant negative noise correlations were proportionally fewer (14% of 6452 pairs), perhaps reflecting a floor effect for most neurons given the low overall mean rates of the full population (Supplemental Figure 4). Example data are given in Figures 5B,C. The left panels of each depict color-mapped firing rate vectors for two sets of neurons grouped according to the aforementioned categorization scheme. The upper right panel of each depicts the mean rate vector for each group, their correlation being negative in one case (Figure 5B) and positive in the other (Figure 5C). Lower right panels in each depict the actual noise correlations for the subsets of neuron pairs recorded simultaneously (dark blue bars) as well as the mean (±2 *SD*) of the noise correlations obtained for randomizations of trial order (light blue bars). The result suggests that the often-complex patterns of task-phase-specific firing patterns shared amongst different sub-populations of BF neurons reflect a high level of complexity in the organization of inputs to those sub-populations, and/or connectivity among BF neurons themselves.

### Coherent patterning of basal forebrain and posterior parietal cortex neurons

**Figure 6:**
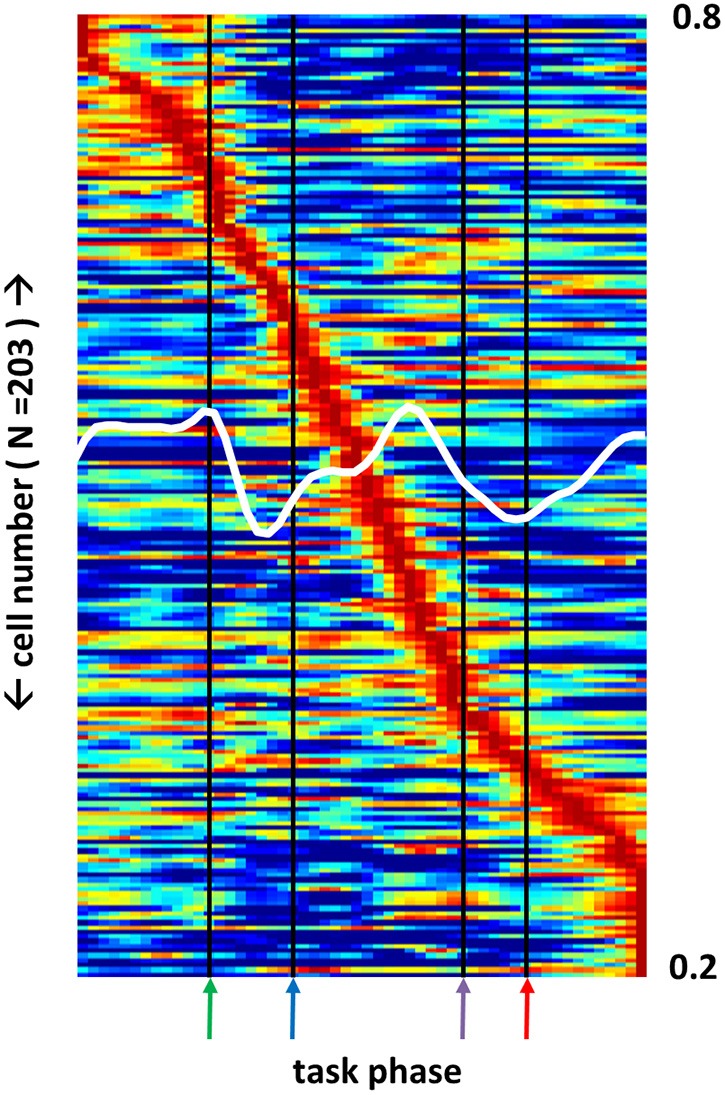
**Posterior parietal cortex neuron sub-populations exhibit firing peaks at all phases of a selective attention task**. Firing rate across all task phases (x-axis) for 203 posterior parietal cortex (PPC) neurons (left y-axis). Each neuron's firing rate is color-mapped as a proportion of its maximum firing rate observed during task performance (blue-red = 0–1). White trace (right y-axis) depicts the mean max-normalized firing rate among all neurons. As for basal forebrain (BF), different PPC neuron populations are maximally active at each task phase, but many fewer neurons exhibit responses to the light flash.

Multiple BF neuron sub-types (GABA, glutamate, ACh) project heavily to regions of cortex and form synapses, in particular, with populations of cortical interneurons that, in turn, are highly interconnected. As a result, specific BF ensemble firing patterns exhibit anatomical connections capable of driving similar patterns among the much larger populations of neurons composing their cortical targets. Projections of BF ACh neurons into posterior parietal cortex impact the ability of animals to increment attention to conditioned stimuli (Bucci et al., 1998). Consistent with these anatomical and behavioral results, a population of posterior parietal cortex (PPC) neurons (*N* = 203) recorded during performance of the same task, exhibited many of the same complex response fields as their BF counterparts (Figure 6).

**Figure 7:**
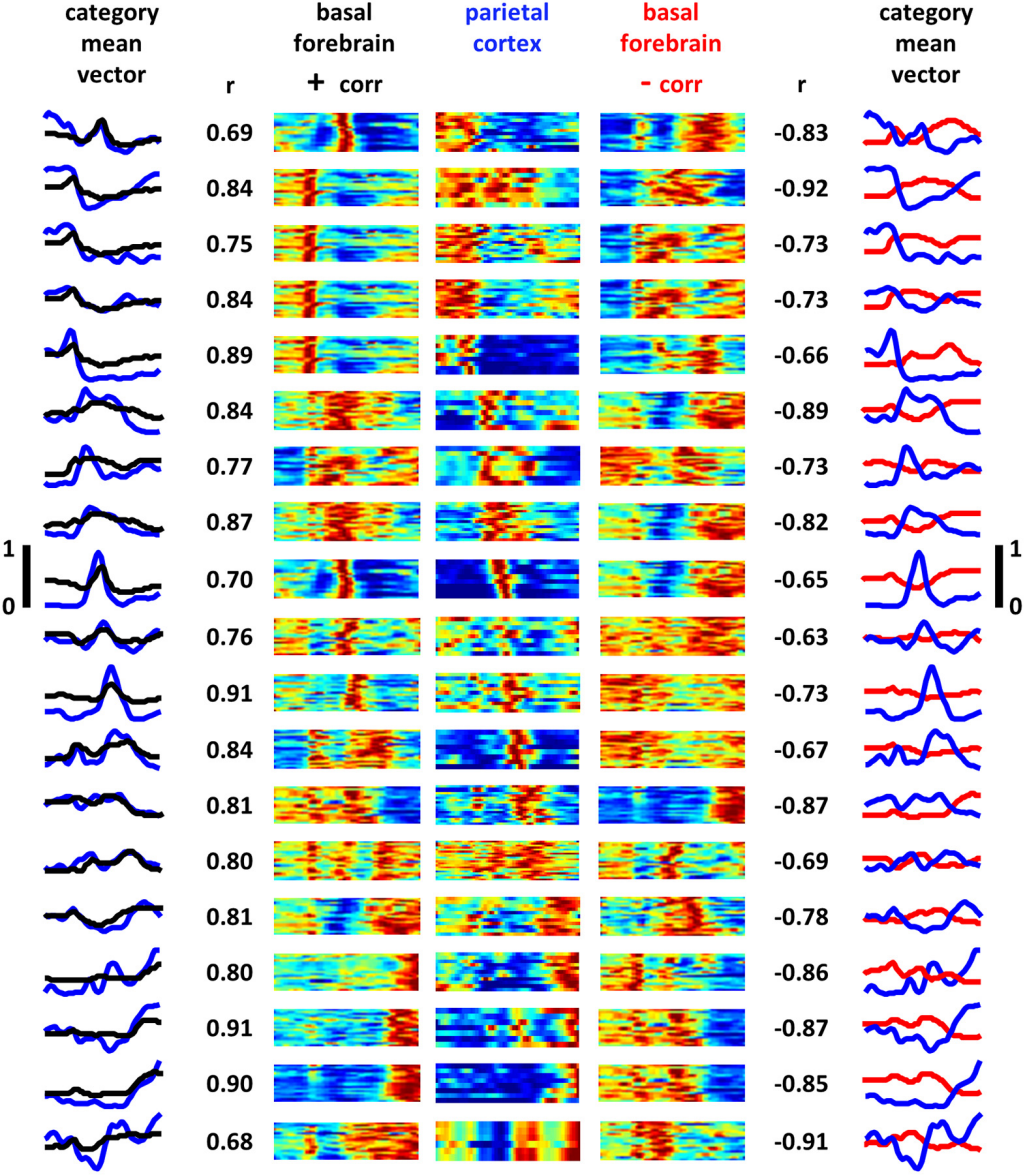
**Posterior parietal cortex task-phase-specific firing patterns parallel those of basal forebrain**. Task-phase-specific firing rate vectors for all recorded PPC neurons (*N* = 203) were grouped into 19 categories according to the method described in Supplemental Figure 3 (see also experimental procedures). The center panels are the full max-normalized firing rate vectors for all task phases for all PPC neurons (y-axis is neurons, x-axis is task phase, blue-red = 0–1). Neighboring color maps to the left and right correspond to full firing rate vectors of all BF neurons for the category of BF neurons whose mean cross-neuron rates bear the greatest overall similarity (left panels) and dissimilarity (right panels) to the associated PPC mean rate. Maximum similarity and dissimilarity were determined through Pearson correlation of the mean cross-neuron firing rate vectors and are given alongside each BF panel. Leftmost panel depicts the mean cross-neuron firing rate vectors for PPC (blue traces) and BF (black traces) neuron populations having maximum similarity. Rightmost panel depicts the same but for categories that are maximally dissimilar (y-axis scales given to left and right of middle panels). For each firing rate pattern observed in basal forebrain, a close match (minimum correlation 0.68) and mismatch (maximum correlation −0.65) are found among populations of PPC neurons.

Applying the same clustering algorithm used to categorize the much larger population of BF neurons, PPC neurons were split into 19 groupings of neurons having similarity in the shape of their full task-phase-specific firing patterns (Figure 7). Remarkably, despite independence in the application of the algorithm to the BF and PPC populations, mean rate vectors for nearly all PPC groups had BF counterparts with highly similar and highly dissimilar patterns. Choosing, for each PPC grouping, the BF grouping with the strongest positive and negative correlation in mean pattern, the mean positive correlation across all groups was 0.81 (±0.07 STD) and the mean negative correlation −0.78 (±0.10 STD). Furthermore, the same noise correlation analysis used for basal forebrain neuron pairs was used to examine BF/PPC cell pairs that were recorded simultaneously. 63 out of 390 (16%) of BF/PPC neuron pairs with positive signal correlations (>0.4) had statistically significant positive noise correlations. In contrast 46 out of 387 (12%) BF/PPC neuron pairs with negative signal correlations (<−0.4) had statistically significant negative noise correlations. Thus, the task-phase-specific activity of PPC neurons takes on similar forms of complex patterning to that of BF neuron populations, indicating that task performance is associated with a series of activity patterns that, for BF and at least one of its efferent targets, run in parallel.

## Discussion

As a brain structure for which normal functioning is of critical importance to diverse cognitive processes, it is remarkable how few studies have examined the dynamics of BF neurons during behavioral task performance. In the present work, large ensemble recordings of BF neurons were obtained during performance of a selective attention task with the goal of filling, to some extent, this gap in knowledge. Defining fundamental properties of BF neural dynamics was enhanced by: (1) utilizing a task having multiple, distinct, and definable event sequences with adequate temporal separation; (2) taking advantage of recent advances in analytical approaches to the sub-typing of complex response fields; and (3) examining to what extent the observed properties of neurons are relevant to the patterns of activity observed in one of its efferent targets.

### Response fields of basal forebrain neurons

A fundamental question at hand in the present study concerns whether the BF neuron population operates more as a unified group, simultaneously impacting all efferent targets when activated, or instead operates as a set of distinct groups that differentially impact their efferent targets across time. The former possibility may, at least to some extent, be the case for other neuromodulatory systems, such as the dopaminergic (Schultz, 1997) and noradrenergic systems (Usher, 1999), where a high percentage of the population is responsive to target stimuli and reward expectation error, respectively. The latter possibility, highly differential responsivity among BF neuron sub-groups, implies heterogeneity in the response fields observed for BF neuron populations. In addition, provided that any given set of sensory, motor, and cognitive states can drive activity in some BF neurons, such heterogeneity could also take the form of generating distinct ensemble firing patterns for all dissimilar phases of a given task.

Application of an objective classification algorithm to the task-phase-specific firing profiles of a large population of neurons produced enhanced visualization of a wide variety of complex firing patterns (Figure 3, Supplemental Figure 3). Most patterns were observed in multiple animals, and all were based on trials associated with uninterrupted behavior prior to and following correct choice-making (Supplemental Figure 1). As a result, the wide array of observed patterns cannot be described as epiphenomenal to peculiarities in how individual animals perform the task. Thus, there is great heterogeneity in the response fields of BF neurons, well beyond what has previously been described, and, indeed, reaching such an extent that distinct ensemble firing patterns were observed for each task phase (Figure 3D).

Basic features of BF neuron task-phase-specific activity patterns are consistent with the idea that the BF neuron population as a whole, by virtue of its heterogeneous anatomy and the complexity of its constituent circuits, is influenced by many different factors and that the influence of these factors is spread unevenly across the population. A large number of neurons did exhibit single peaks in activity in response to light flash onset and reward obtainment, responses emphasized in all prior work (Wilson and Rolls, 1990a,b; Tindell et al., 2004; Lin and Nicolelis, 2008). However, isolated activity peaks were also observed to coincide with nose-poke and the moment when the animal steps up to the center plate upon return from the perimeter. In some cases, isolated peaks in firing were not as easily described as simple sensory or motor responses. One class of neurons, for example, was found to exhibit activity peaks approximately halfway through the return trip to the center plate following nose-poke (Figure 3E, row 3, column 1). Still more evidence of multi-dimensional responsiveness on the part of BF neurons is given by the large population of neurons having multiple peaks in activity, and neurons with activity peaks that extend across several task phases. In all, the BF neuron population appears to maintain a high degree of non-linearity in its responsiveness to the types of stimuli, motor acts, and cognitive processes occurring at different task phases. The observed heterogeneity in response in many ways resembles that described for association cortices (e.g., Cowen et al., 2012; Rigotti et al., 2013). Indeed, a similarly complex set of task-phase-specific patterns was observed for a population of PPC neurons recorded during performance of the same task (Figure 6).

Together, then, the findings support a model in which complexity in the neurochemical identity of BF neurons and spatial specificity in targeting of their efferents is maximally utilized to impact thalamic and cortical processing in a highly spatially specific fashion across time, according to the state of the animal as defined by the current set of sensory inputs, required motor outputs, and task-related cognitive processes. The model is consistent with experimental work demonstrating that different sub-regions of prefrontal cortex can activate different BF neuron sub-groups which, in turn, can differentially impact responsiveness in primary somatosensory and visual cortices (Golmayo et al., 2003). It is also consistent with hypothesized features of BF function based primarily on its input/output connectivity (Bigl et al., 1982; Alheid and Heimer, 1988; Zaborszky and Cullinan, 1991; Groenewegen et al., 1993; Zahm et al., 1996; Zahm, 2006; Zaborszky et al., 2013) In the present task, it is reasonable to assume that relevant cognitive processes include attention to the arena perimeter in the time period prior to a light flash, working memory for the location of the light flash during travel to the arena perimeter, trajectory planning, and encoding of reward. BF neuron sub-groups having activity peaks across task phases associated with each of these processes were observed.

Remarkably, independent classification of task-phase-specific response patterns for a population of PPC neurons yielded sub-groupings for which the mean patterns of activity were highly similar as well as highly dissimilar to one or more BF sub-groups. The PPC of the rat is, as in primates, an association cortex (Krieg, 1946; Ferreira, 1951; Kolb and Walkey, 1987; Reep et al., 1994; Burcham et al., 1997), one that is targeted by efferents from the ventral pallidum sub-region of BF (Kristt et al., 1985; Zaborszky et al., 1997; Bucci et al., 1998; Nelson et al., 2005). Neurons in this structure can exhibit a variety of activity correlations, including those to rather abstract variables such as position in a route (Nitz, 2012) and head orientation relative to the environment (Chen et al., 1994a). Rat PPC neurons also respond to simple sensory stimuli (Broussard et al., 2006), simple motor acts (McNaughton et al., 1994; Nitz, 2006; Whitlock et al., 2012), and specific body postures (Chen et al., 1994b). Given such multimodality in the responsiveness of PPC neurons, the remarkable feature of their task-phase-specific activity patterns in the present work lies not so much in the fact that sub-groups were active at each task phase, but that complexities in their patterns proceed in parallel to those of BF sub-groups (Figures 6, 7). This novel result suggests that the population of active BF neurons at any given time either biases individual cortical neurons toward their task-phase-specific response patterns and/or that BF neuron activity patterns reflect their participation in more macroscopic states of activity that encompass the full set of cortical regions having afferent/efferent connectivity with the BF. Both possibilities are supported by work showing that neurons of the prelimbic sub-region of prefrontal cortex exhibit activity correlates to all phases of a task requiring stimulus detection and choice of an instrumental response, and that such activity correlates are strongly dependent on BF efferents (Gill et al., 2000).

Finally, the categorization algorithm was successful not only in finding BF neuron sub-types that share task-phase-specific activity patterns, but also in revealing pattern opposites. For nearly every sub-grouping of BF neurons generated by the categorization algorithm, a counterpart sub-group having a strongly negatively correlated mean pattern was found (Figure 4). A corollary of this is that for every task phase, there exists a group of BF neurons reaching their activity peak at the same time that another group reaches their activity trough (Figure 3A). Activation/inactivation among such sub-groups appears to be coordinated inasmuch as significant negative trial-by-trial covariance in responses relative to mean rates (i.e., noise correlations) exists for neuron pairs having oppositely-tuned firing patterns (Figure 5).

Given that the neurochemical identity and precise cortical targets of any single BF neuron in the present dataset are unknown, it is difficult to pinpoint the neurophysiological implications of BF sub-groups having opposite firing patterns. It is possible, of course, that populations of activated/inactivated sub-groups do map onto BF neurons differing in their neurochemical identity (i.e., GABAergic vs. glutamatergic vs. acetylcholinergic) and that specific combinations of activity among these neuron types yield specific forms of influence on their efferent targets. In an admittedly simplistic scenario, for instance, GABA and glutamate neurons could reach simultaneous peaks and troughs in activity, yielding amplification of GABAergic inhibition through coincident disfacilitation of excitatory glutamatergic input. Thus, tools to identify the neurochemical identity of extracellularly-recorded BF neurons will undoubtedly be critical in determining the meaning of BF sub-groups having oppositely-tuned activity. Equally important in finding a functional role for pattern opposites is to determine whether the population of neurons exhibiting coincident activity peaks and troughs at any given time have spatially overlapping or non-overlapping efferent targets, either within or across cortical sub-regions, cortical layers, and/or cortical neuron sub-types (e.g., interneurons vs. pyramidal neurons).

BF neurons having correlated mean task-phase-specific firing patterns were also found to have correlated deviations from those means across phases of individual trials (Figure 5). This result could indicate that sub-populations of BF neurons share common input streams. Alternatively, noise correlations could indicate local interconnection among BF neurons or reciprocal connectivity between BF neurons and the brain regions to which they project. The latter two possibilities are supported by: (1) anatomical data describing local collaterals of BF ACh neurons; (2) the existence of BF interneurons (Zaborszky and Duque, 2000); (3) neurophysiological data demonstrating that task-phase-specific activity within the prefrontal cortex, a major source of BF afferents, is itself highly dependent on afferents from the BF (Gill et al., 2000); and (4) the presence of significant correlations in the trial to trial variations in task-phase-specific activity seen for simultaneously recorded BF and PPC neurons.

### Implications for basal forebrain function

With the advent of modern stimulation techniques (e.g., optogenetics) and their potential application to neurological disorders, it is critical to understand how the spatial and temporal resolution of neural dynamics within any given brain region relate to the functional role of that brain region. This is especially the case for the BF given its implication in a wide range of disorders and the powerful impact of its efferents.

To date, the functional role of the BF has been examined primarily through lesion and stimulation studies, complemented only sparingly by recordings of BF neurons in awake, task-performing animals. Most such work has emphasized a prominent role for the BF in arousal (e.g., Buzsáki et al., 1988; Szymusiak et al., 2000; Lee et al., 2004; Goard and Dan, 2009; Hassani et al., 2009), attention (Chiba et al., 1995), learning (Wilson and Rolls, 1990a,b), and/or features of cortical sensory and motor representation and responsiveness (Parikh et al., 2007; Lin and Nicolelis, 2008; Bhattacharyya et al., 2013). The present work was designed to reveal a potentially larger role for the BF in representing the structure of a task, by obtaining multiple single neuron recordings geared toward defining fundamental properties of BF dynamics, such as their response fields, the relation of their dynamics to those of their cortical targets, and their tendency to form groups having positively or negatively correlated responses. Nevertheless, the results have relevance to the issue of the functional role(s) played by the BF in cognition, particularly its role in attention.

In the present study, animals were required to adopt positions and head orientations within a certain range in order to trigger a light flash. As a result, the time period leading up to the light flash can reasonably be considered one associated with heightened attention to the visual field in anticipation of an expected stimulus. While the array of observed task-phase-specific activity patterns in the present study is not inconsistent with a role for the BF in such processes, it is also not suggestive of a role limited to this form of attention. Among the full population of BF neurons, mean activity rates were actually lowest during this time period, and associated with peak activity in only a few neurons. Instead, the time period just after light flash was associated with peak firing in the largest proportion of neurons as well as the highest mean rate across the full population. Thus, to the extent that maximal firing among the population of BF neurons is revealing of its functional role, it appears that the BF plays a major role in initiating and maintaining behavioral responses to relevant stimuli, a conclusion consistent with the results of published recording studies (Parikh et al., 2007; Lin and Nicolelis, 2008).

An alternative view of BF function, one more directly consistent with both the complexity of its anatomical features and electrophysiological dynamics, is that, at any given time, the currently active set of BF neurons yields enhanced responsiveness among the subset of efferent targets reached by those neurons. In this scenario, the greater proportion of neurons having activity peaks in association with the light flash and reward obtainment simply reflects the need for enhanced responsivity across a larger set of BF efferent targets. The set of BF neurons activated at any given task phase is presumably tuned to the associated sensory, motor, and cognitive demands of the task. The observation of neurons with multiple task-phase-specific activity peaks and neurons with activity peaks persisting across multiple task phases perhaps reflects the fact that effective task performance requires enhancement of responsiveness in the target fields of those neurons over the associated task phases. This interpretation is consistent with the work of Zaborszky (Golmayo et al., 2003; Zaborszky et al., 2013) demonstrating that differential activation of neighboring BF neurons with efferents reaching different cortical targets yields site-specific enhancement of responses to sensory stimuli. Furthermore, it is more generally consistent with: (1) the results of studies demonstrating enhanced responses of cortical neurons following BF stimulation or exposure to ACh or ACh receptor agonists (Zhu and Waite, 1998; Disney et al., 2007; Broussard et al., 2009; Goard and Dan, 2009; Takata et al., 2011; Ma and Luo, 2012; Soma et al., 2013); (2) the observed changes in cortical sensory and motor representations associated with the pairing of BF activity and specific sensory stimuli or motor acts (Kilgard and Merzenich, 1998; Dimyan and Weinberger, 1999; Conner et al., 2003); and (3) the wide array of impairments in cognition associated with BF lesions in humans and animals (Szymusiak and McGinty, 1986; Biggan et al., 1991; Roberts et al., 1992; Muir et al., 1993; Baxter et al., 1995; Chiba et al., 1995; Leanza et al., 1996; Stoehr et al., 1997; Zhu and Waite, 1998; McGaughy et al., 2002).

### Conflict of interest statement

The authors declare that the research was conducted in the absence of any commercial or financial relationships that could be construed as a potential conflict of interest.
