# Supplementary figures and images for: Task-phase-specific dynamics of basal forebrain neuronal ensembles
Source: Front Syst Neurosci. 2014 Sep 24;8:174. doi: 10.3389/fnsys.2014.00174 (PMC4173808; doi:10.3389/fnsys.2014.00174)

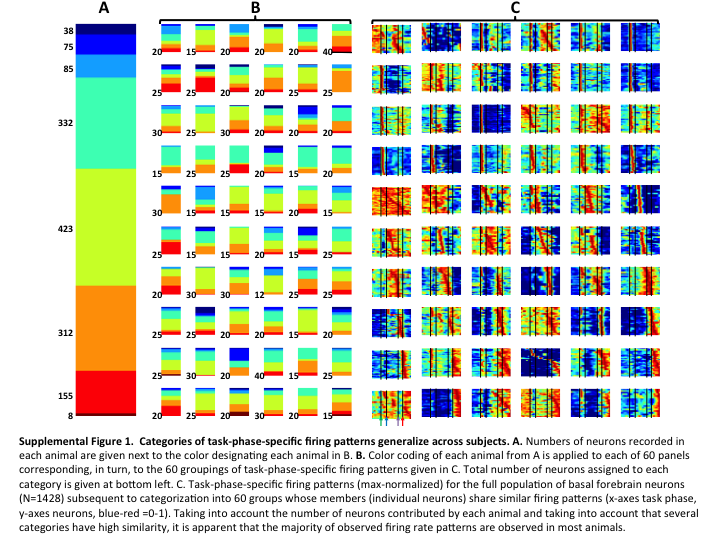

Supplement: Supplementary file 1 [file Image1.TIFF]

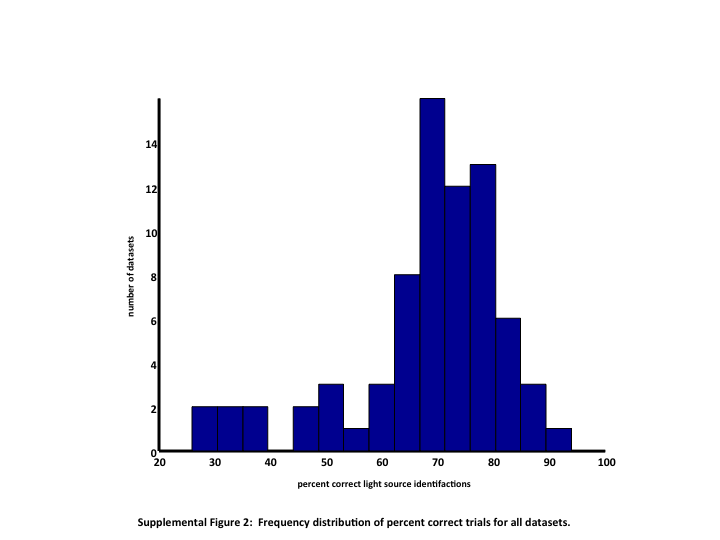

Supplement: Supplementary file 2 [file Image2.TIFF]

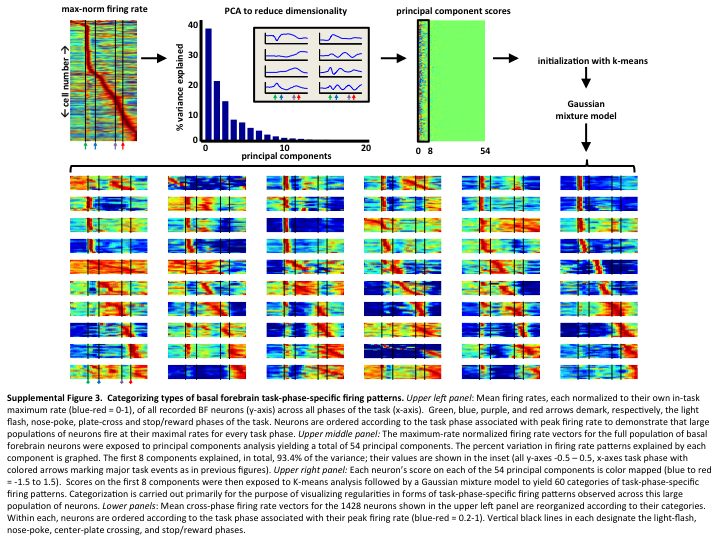

Supplement: Supplementary file 3 [file Image3.TIFF]

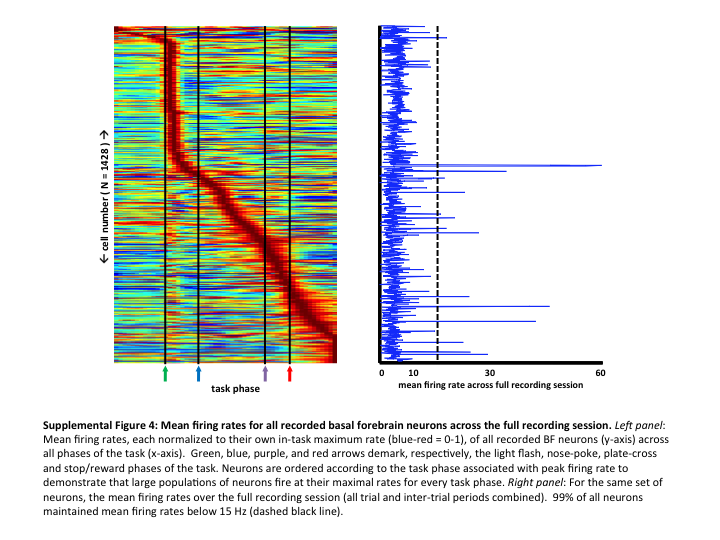

Supplement: Supplementary file 4 [file Image4.TIFF]
