# Supplementary material for: Task-phase-specific dynamics of basal forebrain neuronal ensembles
Source: Front Syst Neurosci. 2014 Sep 24;8:174. doi: 10.3389/fnsys.2014.00174 (PMC4173808; doi:10.3389/fnsys.2014.00174)
Supplement: Supplementary file 5 [file Table1.PDF]

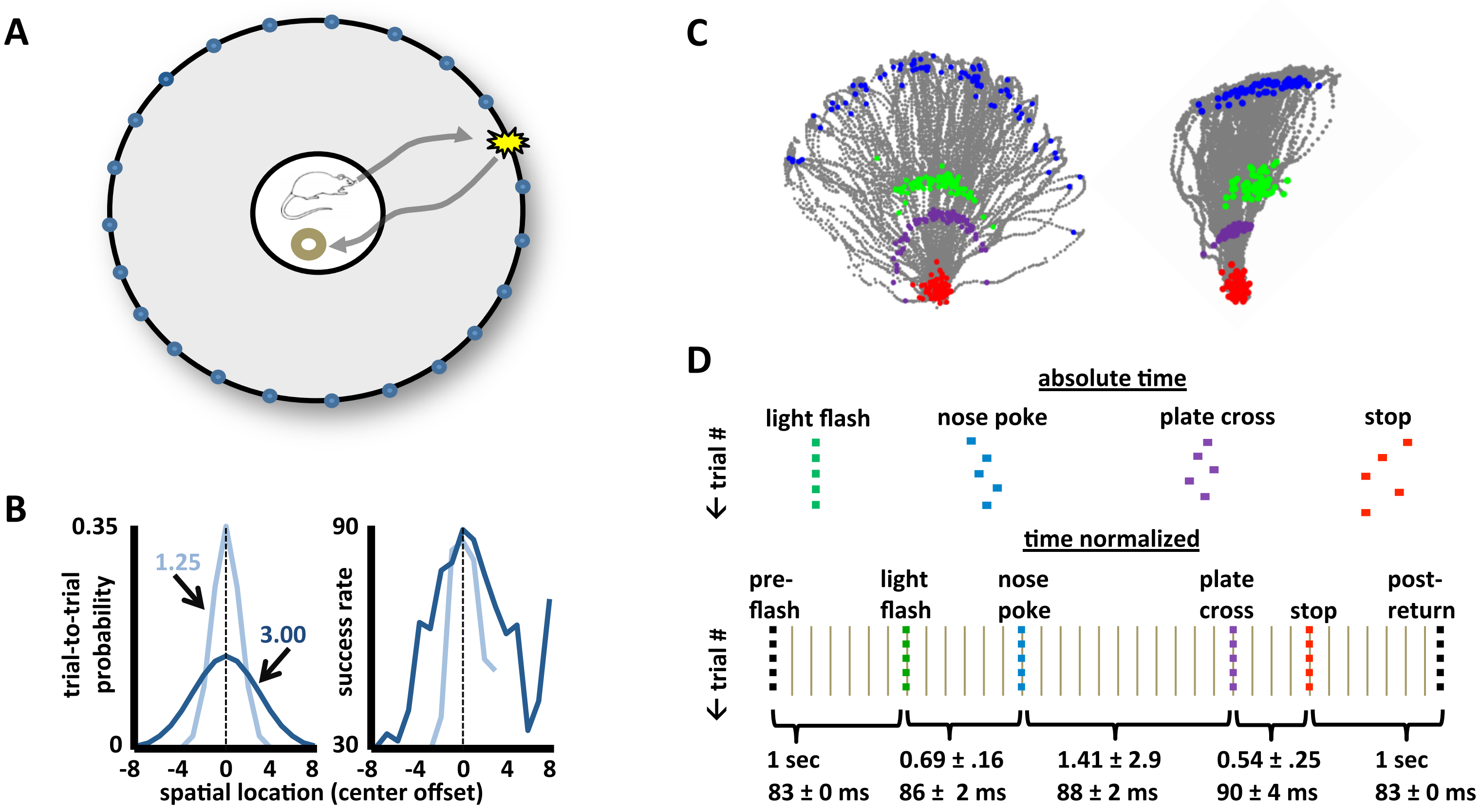

A
B
C
D
0.35
0
trial-to-trial probability
1.25
3.00
90
30
success rate
-8
-4
0
4
8
spatial location (center offset)
absolute time
light flash
nose poke
plate cross
stop
trial #
time normalized
pre-flash
light flash
nose poke
plate cross
stop
post-return
1 sec
83 ± 0 ms
0.69 ± .16
86 ± 2 ms
1.41 ± 2.9
88 ± 2 ms
0.54 ± .25
90 ± 4 ms
1 sec
83 ± 0 ms

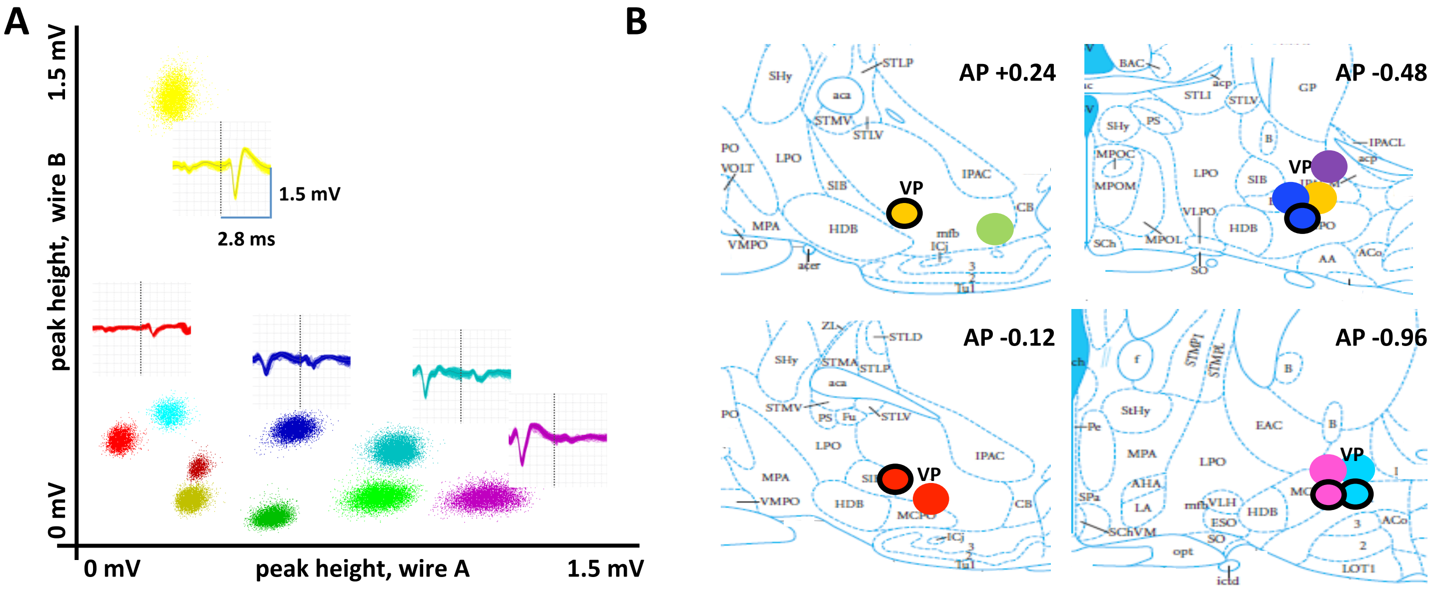

A
1.5 mV
peak height, wire B
0 mV
1.5 mV
2.8 ms
0 mV
peak height, wire A
1.5 mV
B
AP +0.24
AP -0.48
AP -0.12
AP -0.96
VP
VP
VP
VP
SHy
aca
STLP
STMV
STLV
PO
VOLT
LPO
SIB
IPAC
HDB
MPA
VMPO
mfb
ICj
CB
GP
BAC
acp
STLI
IPACL
MPOC
MPOM
VLPO
MPOL
SCh
AA
ACo
SO
STLD
STMA
PS
Fu
MCPO
f
StHy
Pe
EAC
AHA
LA
VLH
ESO
opt
LOT1

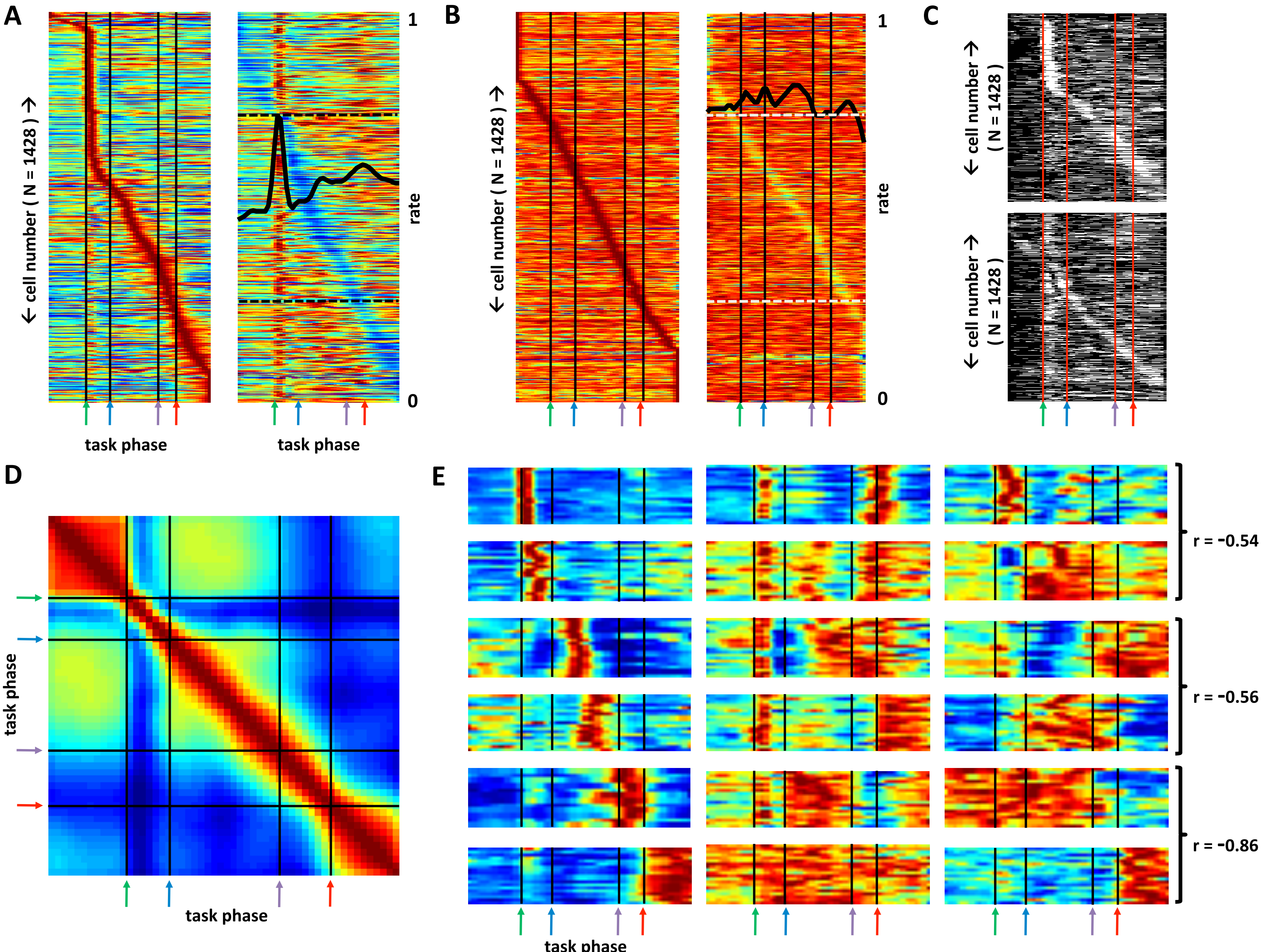
A
B
C
D
E
cell number (N = 1428)
task phase
rate
1
0
r = -0.54
r = -0.56
r = -0.86

A

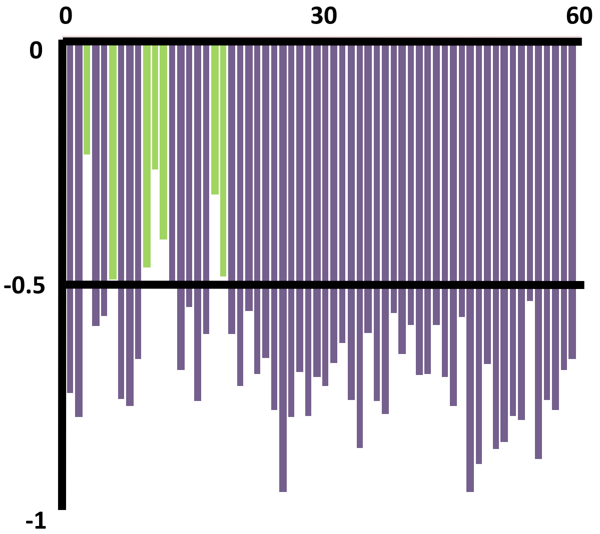

B

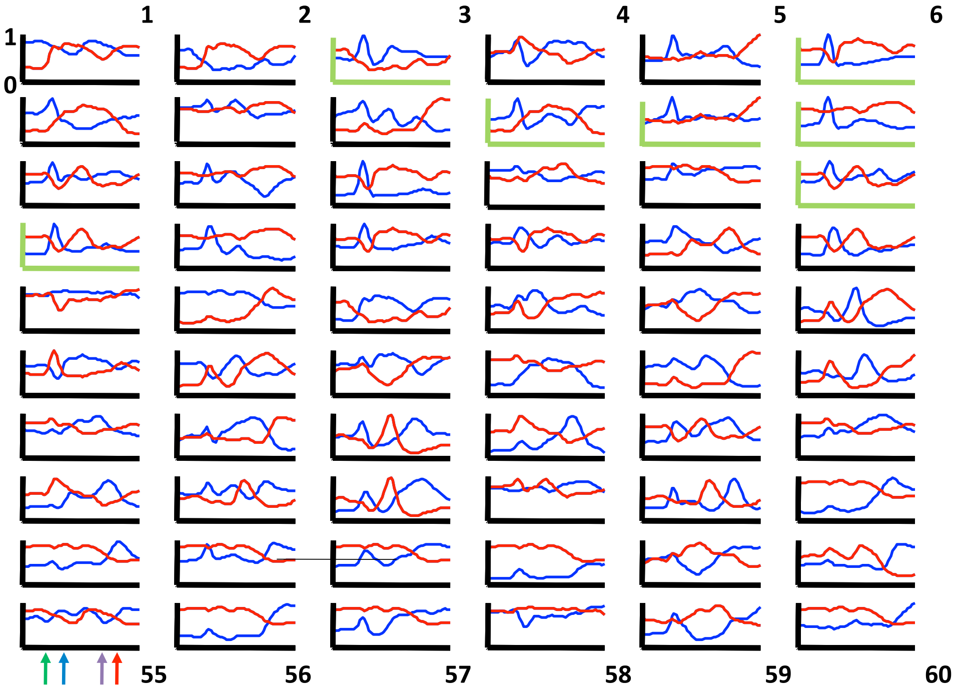

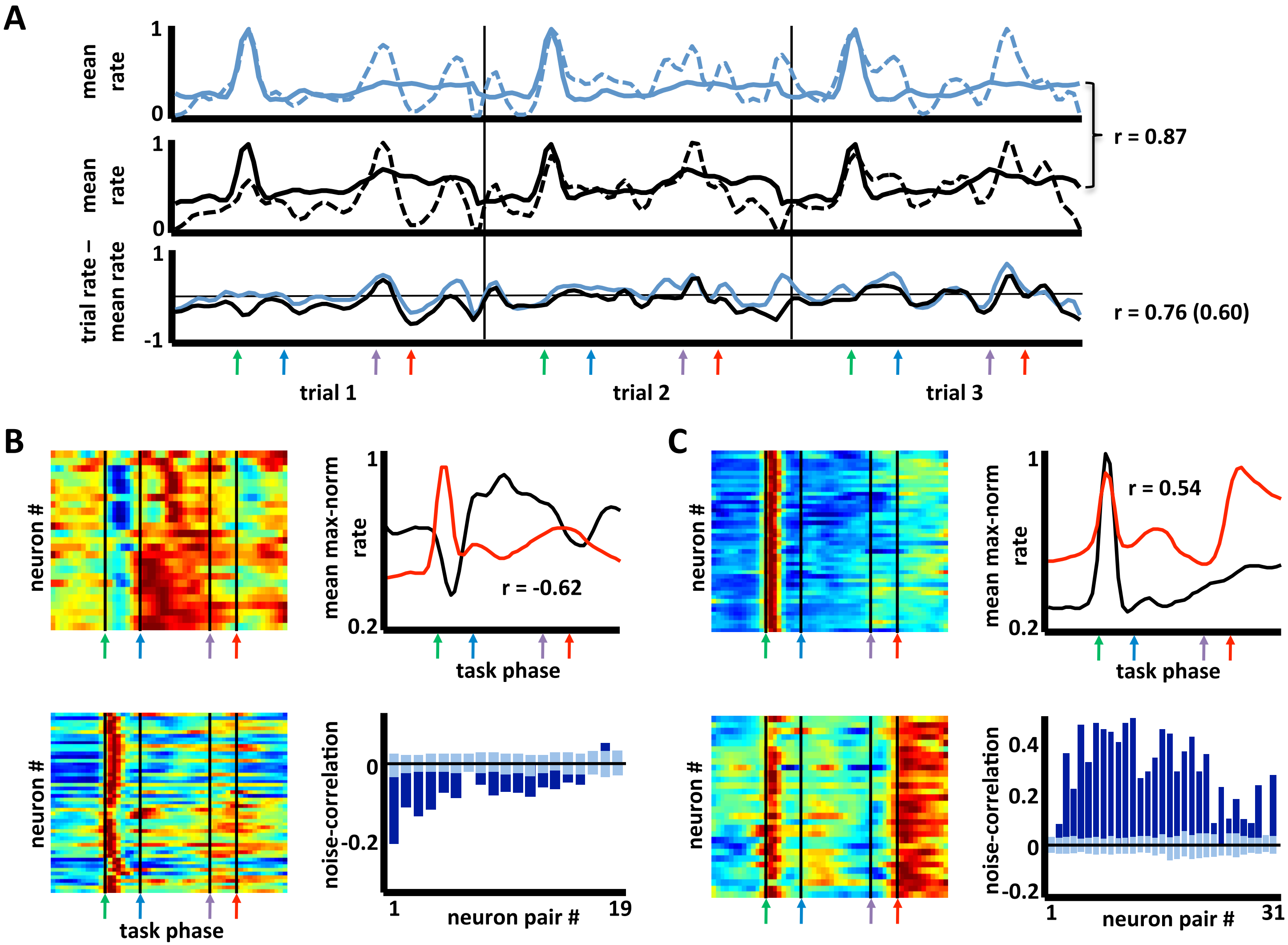

A
mean rate
1
0
r = 0.87
trial rate – mean rate
-1
r = 0.76 (0.60)
trial 1
trial 2
trial 3
B
neuron #
mean max-norm rate
0.2
r = -0.62
task phase
noise-correlation
-0.2
neuron pair #
19
C
r = 0.54
0.4
31

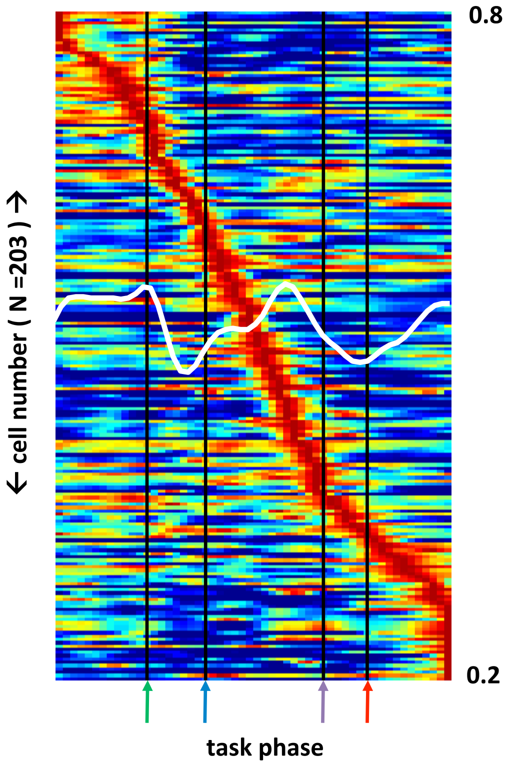

0.8
0.2
← cell number (N =203) →
task phase

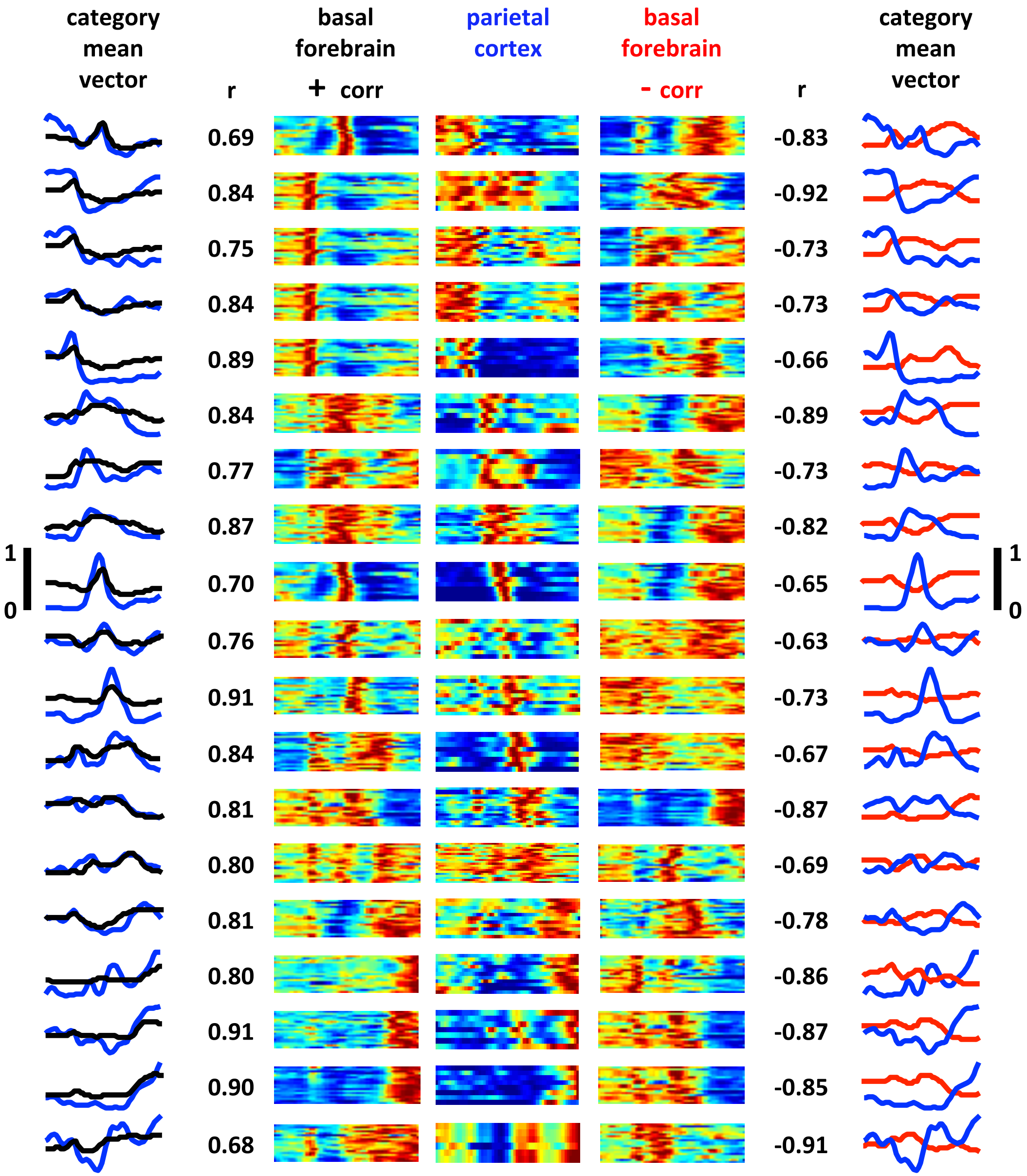

category mean vector
r
basal forebrain + corr
parietal cortex
basal forebrain - corr
r
category mean vector
0.69
0.84
0.75
0.84
0.89
0.84
0.77
0.87
0.70
0.76
0.91
0.84
0.81
0.80
0.81
0.80
0.91
0.90
0.68
-0.83
-0.92
-0.73
-0.73
-0.66
-0.89
-0.73
-0.82
-0.65
-0.63
-0.73
-0.67
-0.87
-0.69
-0.78
-0.86
-0.87
-0.85
-0.91
1
0
1
0

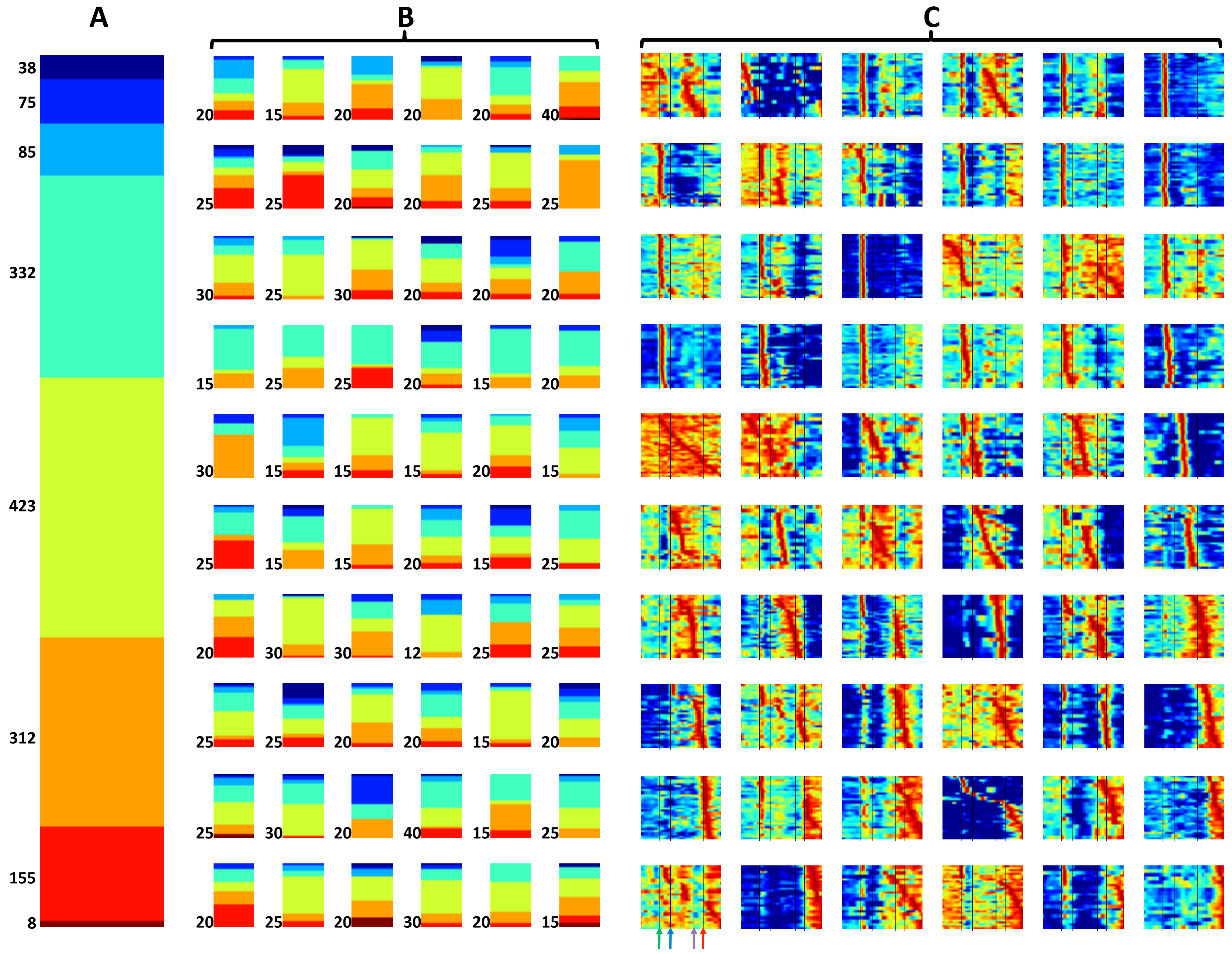
A
B
C
38
75
85
332
423
312
155
8
20 15 20 20 20 40
25 25 20 20 25 25
30 25 30 20 20 20
15 25 25 20 15 20
30 15 15 15 20 15
25 15 15 20 15 25
20 30 30 12 25 25
25 25 20 20 15 20
25 30 20 40 15 25
20 25 20 30 20 15

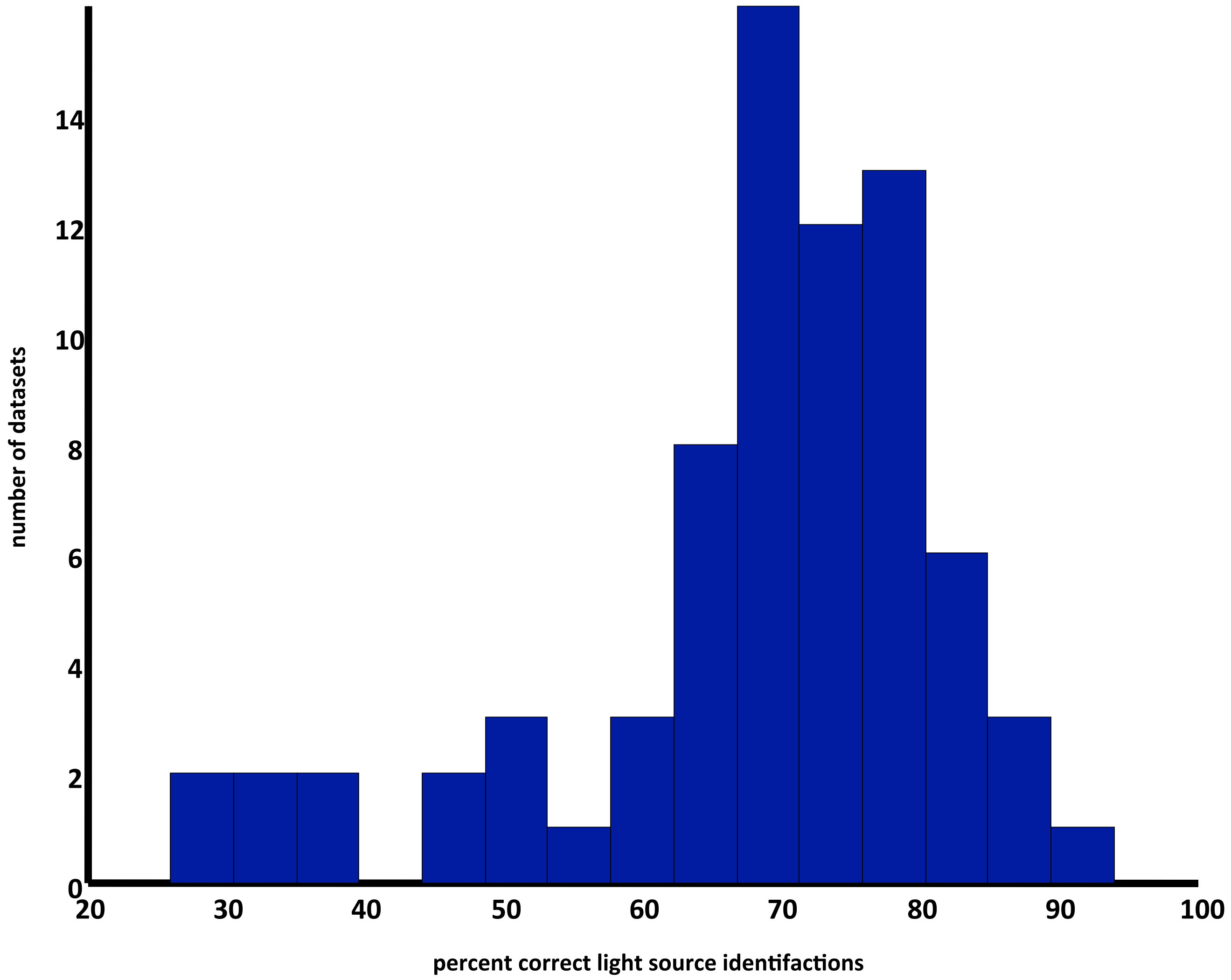
14
12
10
8
6
4
2
0
number of datasets
20
30
40
50
60
70
80
90
100
percent correct light source identifactions

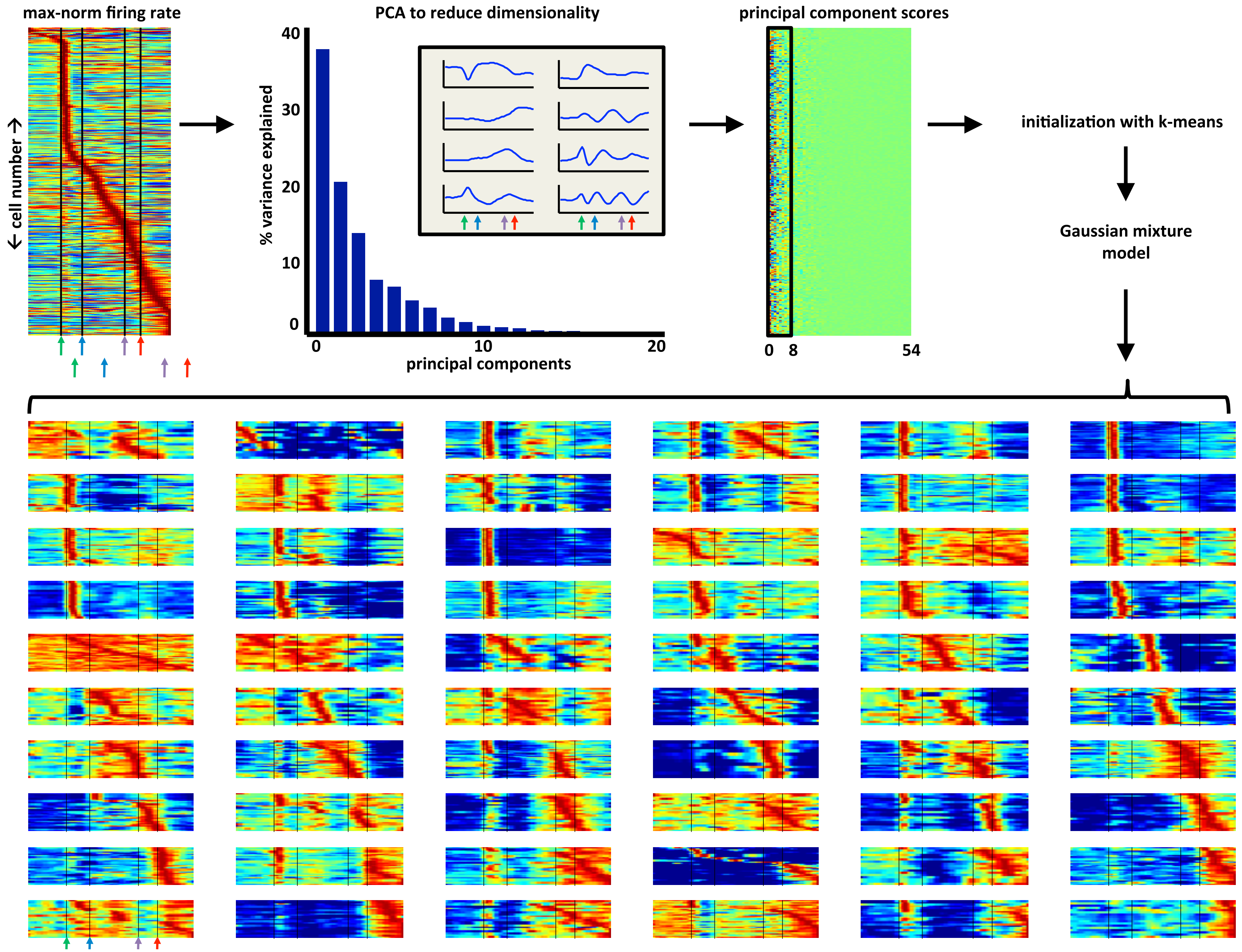
max-norm firing rate
cell number
PCA to reduce dimensionality
% variance explained
40
30
20
10
0
0
10
20
principal components
principal component scores
0
8
54
initialization with k-means
Gaussian mixture model

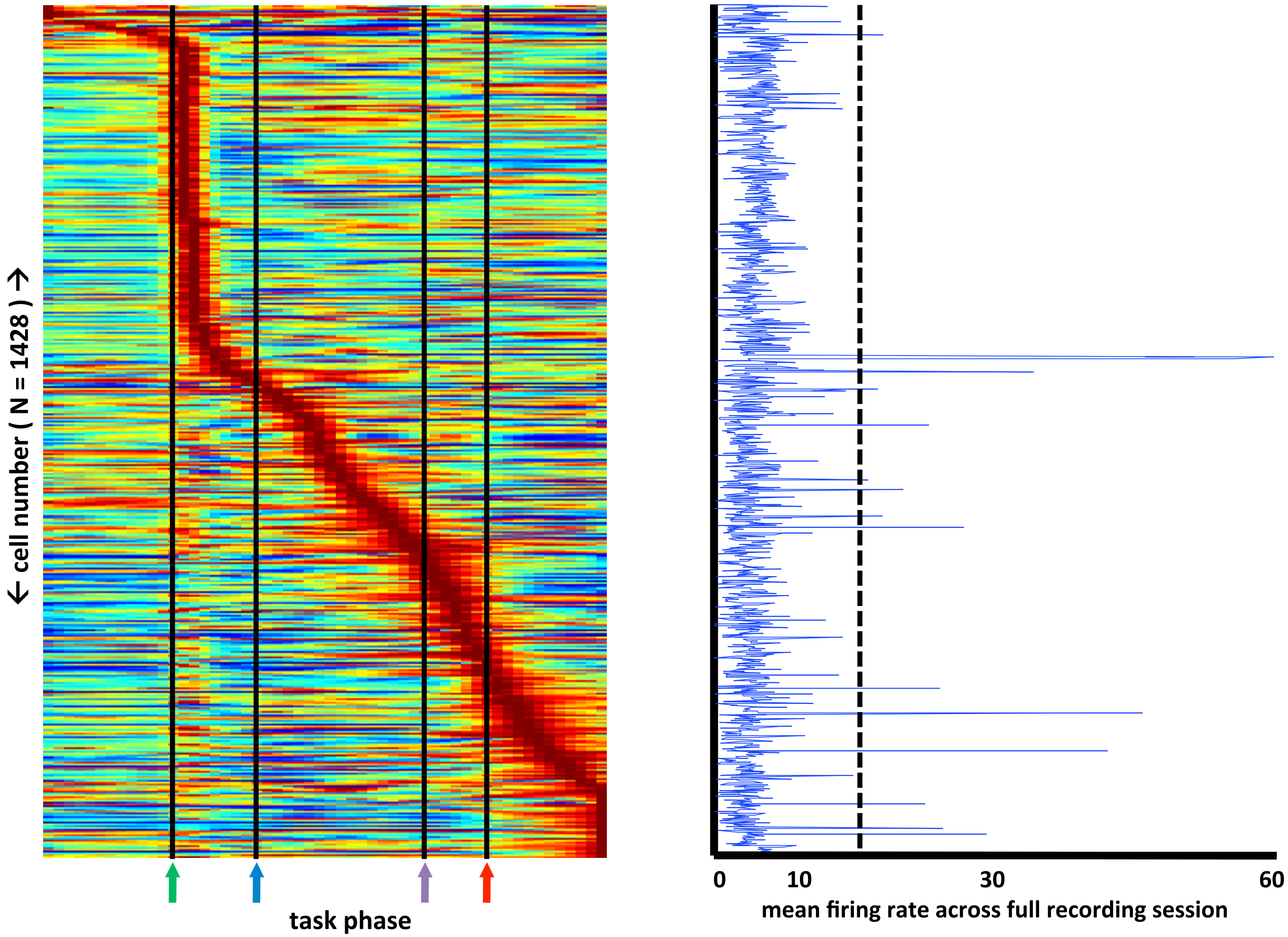

← cell number (N = 1428) →
task phase
0
10
30
60
mean firing rate across full recording session

	RECORDING	ANIMAL	CELL COUNT	DISTRIBUTION
	1	AA8	11	3
AA8	2	AA8	13	3
	3	AA8	9	3
	4	AA8	5	3
	5	DN3	23	1.25
DN3	6	DN3	7	1.25
	7	DN3	30	1.25
	8	DN3	15	3
	9	NS1	4	3
	10	NS1	4	1.25
	11	NS1	4	1.25
	12	NS1	11	1.25
	13	NS1	5	1.25
	14	NS1	12	1.25
	15	NS1	13	1.25
	16	NS1	5	1.25
NS1	17	NS1	4	1.25
	18	NS1	3	1.25
	19	NS1	1	1.25
	20	NS1	1	1.25
	21	NS1	2	1.25
	22	NS1	2	1.25
	23	NS1	2	1.25
	24	NS1	8	3
	25	NS1	4	1.25
	26	NS2	19	1.25
	27	NS2	26	1.25
	28	NS2	29	1.25
	29	NS2	45	1.25
	30	NS2	66	1.25
NS2	31	NS2	48	1.25
	32	NS2	35	3
	33	NS2	34	3
	34	NS2	30	3
	35	NS3	15	1.25

RECORDING	ANIMAL	CELL COUNT	DISTRIBUTION	
35	NS3	15	1.25	
36	NS3	37	1.25	
37	NS3	36	1.25	
38	NS3	51	3	
39	NS3	53	3	
40	NS3	36	3	NS3
41	NS3	41	3	
42	NS3	53	1.25	
43	NS3	47	1.25	
44	NS3	26	1.25	
45	NS3	18	3	
46	NS3	10	3	
47	NS4	43	3	
48	NS4	25	3	
49	NS4	36	3	
50	NS4	23	3	
51	NS4	21	1.25	
52	NS4	21	1.25	
53	NS4	23	1.25	
54	NS4	22	1.25	NS4
55	NS4	25	1.25	
56	NS4	18	1.25	
57	NS4	11	1.25	
58	NS4	3	1.25	
59	NS4	6	1.25	
60	NS4	6	1.25	
61	NS4	12	1.25	
62	NS5	9	1.25	
63	NS5	15	1.25	
64	NS5	18	1.25	
65	NS5	14	1.25	
66	NS5	12	1.25	
67	NS5	14	1.25	NS5
68	NS5	14	1.25	
69	NS5	13	1.25	
70	NS5	11	1.25	
71	NS5	10	1.25	
72	NS5	9	1.25	
73	NS5	16	1.25	
74	NS8	8	3	NS8
